# Differentially profiling the low-expression transcriptomes of human hepatoma using a novel SSH/microarray approach

**DOI:** 10.1186/1471-2164-7-131

**Published:** 2006-05-31

**Authors:** Yi-Shin Pan, Yun-Shien Lee, Yung-Lin Lee, Wei-Chen Lee, Sen-Yung Hsieh

**Affiliations:** 1 Liver Research Unit, Chang Gung Memorial Hospital, Tao-Yuan, Taiwan; 2 Genomic Medicine Core Laboratory, Chang Gung Memorial Hospital, Tao-Yuan, Taiwan; 3 Clinical Proteomics Center, Chang Gung Memorial Hospital, Tao-Yuan, Taiwan; 4 Department of General Surgery, Chang Gung Memorial Hospital, Tao-Yuan, Taiwan; 5 Chang Gung University School of Medicine, Tao-Yuan, Taiwan; 6 Department of Biotechnology, Ming Chuan University, Tao-Yuan, Taiwan

## Abstract

**Background:**

The main limitation in performing genome-wide gene-expression profiling is the assay of low-expression genes. Approaches with high throughput and high sensitivity for assaying low-expression transcripts are urgently needed for functional genomic studies. Combination of the suppressive subtractive hybridization (SSH) and cDNA microarray techniques using the subtracted cDNA clones as probes printed on chips has greatly improved the efficiency for fishing out the differentially expressed clones and has been used before. However, it remains tedious and inefficient sequencing works for identifying genes including the great number of redundancy in the subtracted amplicons, and sacrifices the original advantages of high sensitivity of SSH in profiling low-expression transcriptomes.

**Results:**

We modified the previous combination of SSH and microarray methods by directly using the subtracted amplicons as targets to hybridize the pre-made cDNA microarrays (named as "SSH/microarray"). mRNA prepared from three pairs of hepatoma and non-hepatoma liver tissues was subjected to the SSH/microarray assays, as well as directly to regular cDNA microarray assays for comparison. As compared to the original SSH and microarray combination assays, the modified SSH/microarray assays allowed for much easier inspection of the subtraction efficiency and identification of genes in the subtracted amplicons without tedious and inefficient sequencing work. On the other hand, 5015 of the 9376 genes originally filtered out by the regular cDNA microarray assays because of low expression became analyzable by the SSH/microarray assays. Moreover, the SSH/microarray assays detected about ten times more (701 vs. 69) HCC differentially expressed genes (at least a two-fold difference and P < 0.01), particularly for those with rare transcripts, than did the regular cDNA microarray assays. The differential expression was validated in 9 randomly selected genes in 18 pairs of hepatoma/non-hepatoma liver tissues using quantitative RT-PCR. The SSH/microarray approaches resulted in identifying many differentially expressed genes implicated in the regulation of cell cycle, cell death, signal transduction and cell morphogenesis, suggesting the involvement of multi-biological processes in hepato-carcinogenesis.

**Conclusion:**

The modified SSH/microarray approach is a simple but high-sensitive and high-efficient tool for differentially profiling the low-expression transcriptomes. It is most adequate for applying to functional genomic studies.

## Background

Microarray is a powerful technique for simultaneously determining the expression of thousands of genes [1-3]. Such studies can quickly yield a genome-wide description of mRNA expression, called transcriptomes, in a given cell or tissue at a given physiologic or pathologic condition [4]. Even though, one of the main challenges in such genome-wide gene-expression profiling is the difficult inspection of genes with rare transcripts.

On the other hand, PCR-based suppressive subtractive hybridization (SSH) techniques are highly sensitive for identifying differences in gene expression [5-9]. However, the potentials of SSH in assaying dynamic changes of gene expression in minute levels have never been addressed before. In addition, SSH techniques are also restricted in terms of limited specificity and difficulties in identifying enriched genes. Combining the SSH technique with high-throughput screening of the harvested clones through the use of cDNA microarrays could greatly reduce the tedious work for northern blot analysis, as well as the likelihood of false-positive clones enriched via SSH [10]. Such combined approaches by printing the clones obtained from the SSH amplicones on chips have been successfully used for profiling the differentiation of gene expression [10-13]. Nevertheless, in such approaches, the genes of the subtracted clones remain to be sequenced for identification, and a large portion of redundancy in the enriched amplicons must also be identified. Moreover, since the targets used to hybridize the amplicon clones printed on chips were the un-enriched cDNA pools rather than the subtracted, enriched clones, such approaches would not increase the sensitivity for detection of the low-expression genes.

Herein, we report our modifications using the subtracted amplicons as the targets to hybridize the pre-prepared microarray chips for SSH/microarray analysis. Since all of the probes on the microarray chips have been well characterized, the hundreds and thousands of genes in the subtracted amplicons can be determined by a single hybridization. Moreover, the relative expression status between the compared tissues for each gene can be augmented and easily determined by combining the use of the targets prepared both from forward and reverse subtractions of SSH. We named this modified approach "SSH/microarray" and used human hepatocellular carcinoma as a model to demonstrate the feasibility of this approach.

## Results

### The SSH/cDNA microarray versus regular cDNA microarray approaches

**Figure 1:**
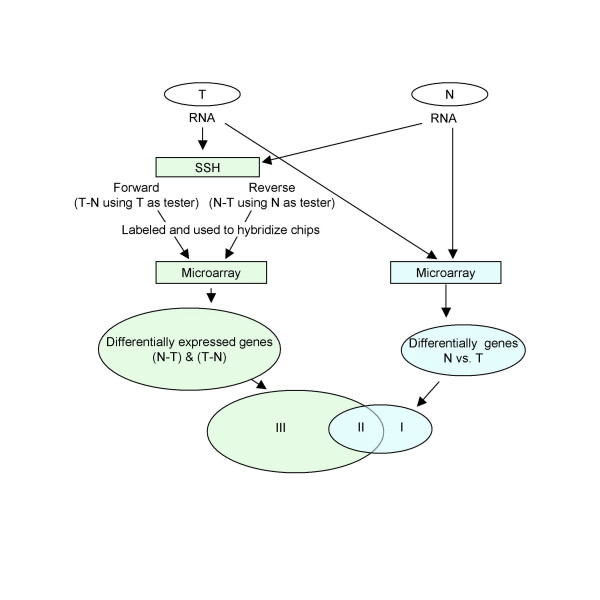
**Experimental flowchart**. Messenger RNA was prepared from hepatoma (T) and the corresponding non-hepatoma liver tissues (N), and then subjected to 1) PCR-based suppressive subtractive hybridization (SSH) followed by using the resulted subtracted cDNA libraries as targets for cDNA microarray analysis (the SSH/microarray), and 2) conventional cDNA microarray analysis. SSH was performed in both the forward (T as tester) and reverse (N as tester) direction to enrich up-regulated (T-N amplicon) as well as down-regulated transcriptomes (N-T amplicon) in human hepatoma, respectively. The two subtracted amplicons were labeled with fluorescent cy-dyes as targets for microarray analysis, in which dye-swapping approaches were used. The results thus obtained were then compared to those obtained from the conventional cDNA microarray assays. The differentially expressed genes were categorized into three groups: I) only detected by conventional cDNA microarray approaches, II) identified by both conventional cDNA microarray and SSH/microarray approaches, and III) only obtained from SSH/microarray assays. The results were further confirmed by qRT-PCR in 6 genes randomly selected from group III differentially expressed genes.

To conduct the comparative transcriptomic studies on human hepatoma particularly for the genes at low-expression levels, we set out an approach as shown in Figure 1. We used the PCR-based suppressive subtractive hybridization to enrich the differentially expressed cDNA clones between human hepatoma and non-hepatoma liver tissues. The SSH was conducted both forwards using RNA prepared from hepatoma and non-hepatoma liver tissues as tester and driver, respectively, to yield the hepatoma (T-N) subtracted amplicons, and reversely using the mRNA prepared from non-hepatoma and hepatoma liver tissues as tester and driver respectively to yield the non-hepatoma (N-T) subtracted amplicons as well. Instead of the previously reported combination with cDNA microarray approaches, in which clones in the subtracted cDNA libraries were cloned and then printed on chips, we directly labeled the subtracted T-N and N-T amplicons and then used as targets to hybridize the pre-made microarray chips printed with the known 14,811 cDNA clones. We named this modified approach as "SSH/microarray". The same tissue RNA pairs and cDNA microarray chips were also used for the regular cDNA microarray assays for comparison. Since human hepatoma generally occurs in patients of chronic hepatitis with/without cirrhosis, to identify the genes implicated in the common biological processes leading to hepatocellular carcinogenesis, we selected three hepatoma patients with different underlying liver disease. The first was with chronic hepatitis C and cirrhosis, the second had chronic hepatitis B and cirrhosis, and the third had chronic hepatitis B but no clinical or histological evidence of cirrhosis.

**Figure 2:**
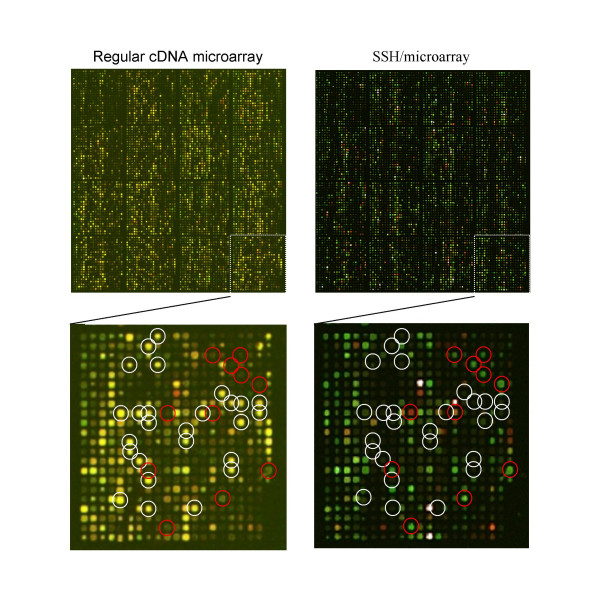
**The representative results of the regular cDNA microarray vs. SSH/microarray assays**. RNA were prepared from the hepatoma and para-hepatoma liver tissues of a patient with HCC. Left panels are the representative results obtained via the regular cDNA microarray assays using the Cy *5 *and Cy3 labeled aRNA generated from hepatoma and non-hepatoma liver tissues, respectively. Right panels are the representative results obtained via the SSH/microarray assays using Cy3 and Cy5 to label the forward and reverse subtracted amplicons, respectively. Lower panels are the close views of the upper panels. White circles indicate the clones, which had equal Cy3 and Cy5 hybridization intensities in the conventional cDNA microarray assays but been subtracted away from the subtracted amplicons. Red circles mark the clones, which had low Cy5 and Cy3 hybridization intensities in the conventional cDNA microarray but presented with differential expression in the subtracted amplicons.

Figure 2 demonstrates the representative results obtained from the corresponding regular cDNA microarray and the SSH/microarray assays. Of note, in the SSH/microarray assays the genes in the subtracted T-N and N-T amplicons were readily identified without tremendous sequencing works, which were required for the original SSH and microarray combination assays. In addition, many of the clones, including those of the house-keeping genes, such as *β-actin *and *GAPDH*, with unremarkable difference in hybridization intensities by the regular cDNA microarray assays were efficiently excluded from the subtracted amplicons (Figure 2, indicated by white circles). This indicated the efficiency of subtraction hybridization. Most importantly, many of the clones with hybridization intensity below the evaluation threshold by the regular cDNA microarray approaches (hybridization intensities lower than or close to the noise) became detectable by the SSH/microarray method (Figure 2, indicated with red circles). These observations suggested a more sensitive detection of the low abundance transcripts using the SSH/microarray assays.

### The SSH/microarray approach on low-expression and differentially expressed genes

**Figure 3:**
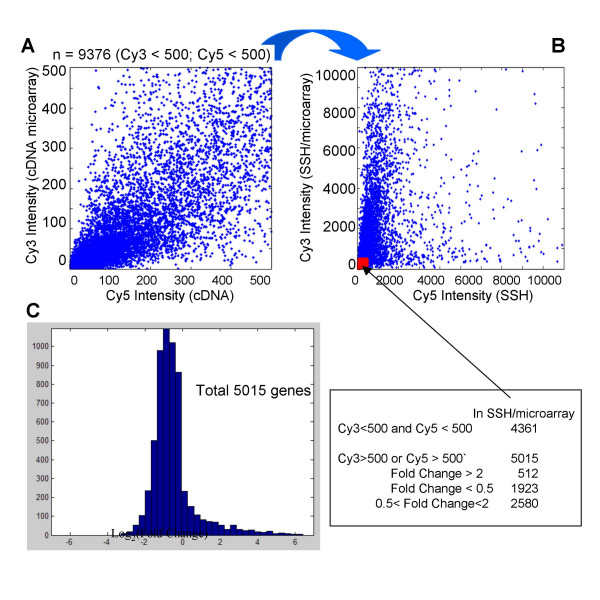
**Detection of differential low-expression transcriptomes by the SSH/microarray**. A) Of the 14811 distinct genes on the chips, 9376 genes had hybridization intensities lower than 500 units (the defined low intensity threshold for exclusion from further microarray analysis) in a representative set of results of the conventional cDNA microarray assay. B)These low-expression genes were then subjected to re-calibration for their Cy3/Cy5 intensities in the SSH/microarray assay and 5015 genes became detectable in SSH assays (blue spots). C) The distribution of these 5015 low-expression genes identified only in the SSH/microarray assays, including 512 and 1923 genes down- and up-regulated in hepatoma tissues, respectively. The data presented in this figure were based on the results derived from patient 1, a case of chronic hepatitis C with cirrhosis and hepatoma.

To further address whether the SSH/microarray approach was able to assay the expression of those genes with rare transcripts, we selectively inspected those genes with low expression. Of the total of 14,811 clones on the chips, 9376 clones that were filtered out for further analyses in the regular cDNA microarray studies due to the low hybridization intensity (intensities <500 unit after the noise subtracted) (Figure 3A) were selected for examination of their hybridization intensity in the SSH/microarray assays (Figure 3B). Of these, 5015 clones were enriched via the SSH/microarray assays and could be analyzed for differential expression (intensities >500 unit after the noise intensity subtracted) (Figure 3B &3C). That is, the SSH/microarray approach allowed about 100% more of the genes, most of which were low abundant, for the subsequent analyses.

Since SSH specifically amplified the difference of gene expression, the SSH/microarray approach should also be able to identify more differentially expressed genes between hepatoma and non-hepatoma liver tissues, which were originally undetectable by the regular cDNA microarray methods. As shown in Fig 3C, of the 5015 low-expression genes identified only by the SSH/microarray assays, we identified additional 512 and 1923 genes with at least a 2-fold decrease and increase in hepatoma tissues, respectively.

**Figure 4:**
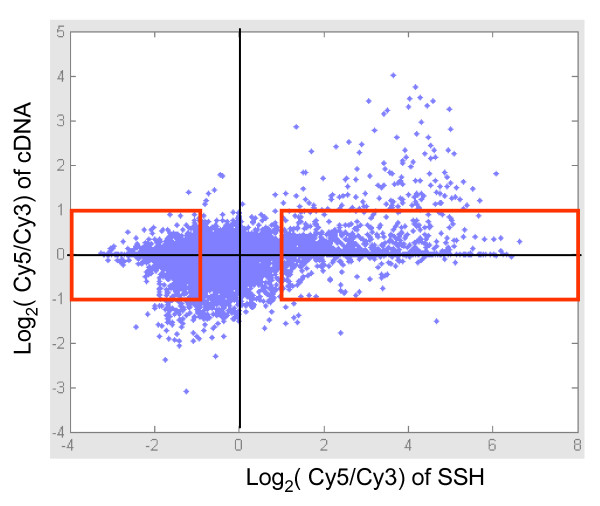
**Comparison of the relatively expression by regular cDNA microarray vs. the SSH/microarray**. The distribution of the ratios of Cy5/Cy3 intensities obtained by the conventional cDNA microarray assays vs. the SSH/microarray assays is shown. Only the clones with significant Cy3 and Cy5 intensities in both assays were included in the analysis. The clones inside the red-boxes were those with their log_2 _ratios < 1 and > -1 via the conventional cDNA microarray assays, while their log_2 _ratios > 1 or < -1 by the SSH/microarray assays. The data presented were based on those derived from patient 1, a case of chronic hepatitis C with cirrhosis and hepatoma.

We further examined the sensitivity for detection of differential expression by both approaches. We compared the ratio of Cy5/Cy3 intensity of each clone obtained from the regular cDNA microarray assays to that obtained from the SSH/microarray assays. As shown in Figure 4, a total of 3028 genes with a ratio lower than 2 folds (-1 < log_2 _< 1) using the cDNA microarray assays had a ratio greater than 2 folds (log_2 _> 1 or < -1) using the SSH/microarray assays (Figure 4, 1091 and 1937 genes indicated by the red box, respectively). That is, using the modified SSH/microarray approaches resulted in much more differentially expressed genes, particularly for those with rare transcripts that using the regular cDNA microarray approaches.

### Identification of differentially expressed genes with low expression in human hepatoma

**Figure 5:**
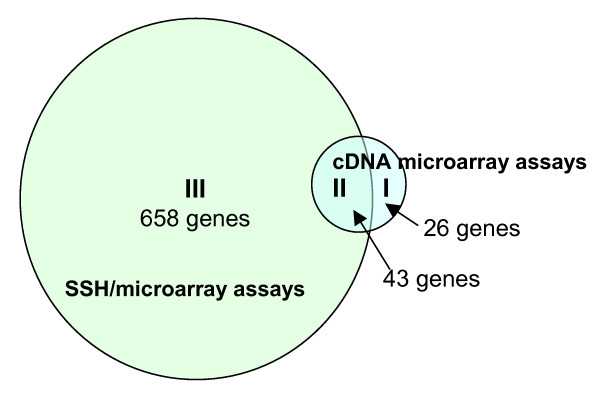
**The differentially expressed genes**. The genes differentially expression in human hepatoma were identified by either only the conventional cDNA microarray assays (26 genes) or SSH/microarray assays (658 genes), or both (43 genes). The results were derived from three pairs of hepatoma liver tissues with at least two-fold difference in gene expression and P < 0.01.

**Figure 6:**
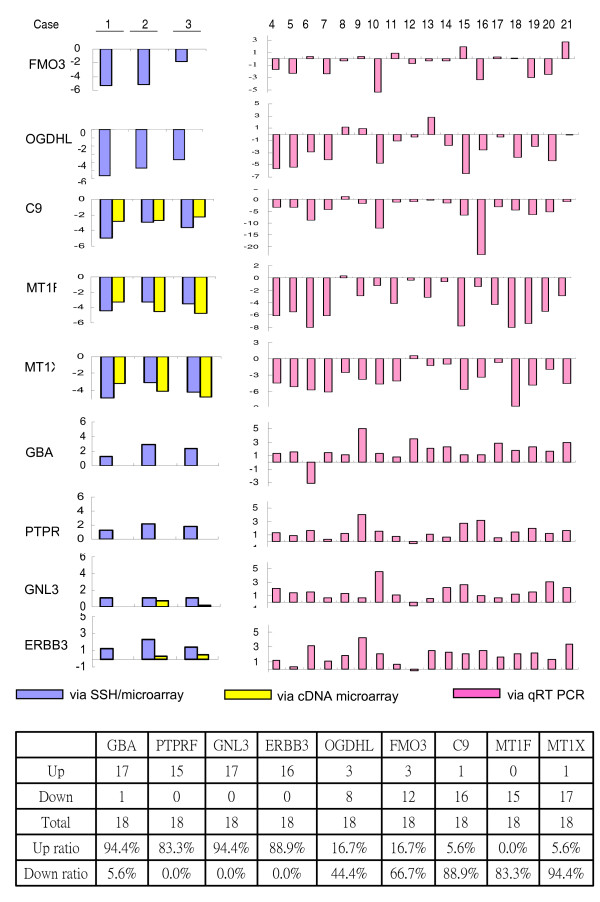
**Quantification of relatively gene expression in human hepatoma**. A total of nine differentially expressed genes including six (*GBA*, *PTPRF*, *GNL3*, *ERBB3*, *OGDHL*, *FMO3 *belonging to Class III) identified only by the SSH/microarray assays and three (*C9*, *MT1F*, *MT1X *belonging to class II) identified by both of the SSH/microarray and regular cDNA microarray assays were assayed for their relative expression between HCC and the corresponding non-HCC liver tissues of 18 patients of hepatoma using real-time semi-quantitative RT-PCR. The results are presented with the log_2 _values of the gene expression ratio's between HCC vs. the matched non-HCC liver tissues. Left panel is the relatively expression level of these genes initially determined by the SSH/microarray and regular cDNA microarray assays, respectively. Of note, the difference of the expression for the six genes belonging to Class III was originally only identified by the SSH/microarray assays, but not by the regular cDNA microarray assays.

A total of 69 and 701 genes differentially expressed in human hepatoma were finally identified (based on the data derived from the three tissue pairs in duplicate with log_2 _> 1 or < -1, and P < 0.01) by the regular cDNA microarray assays and by the SSH/microarray assays, respectively (Figure 5 & see additional file 1). That is, the SSH/microarray assays detected 10-fold more differentially expressed genes, particularly for those low-expression genes, than did the conventional cDNA microarray. To confirm the differential expression of genes with low expression identified by the SSH/microarray assays (including 446 and 255 up- and down-regulated genes, respectively), we quantified the relative expression level of nine randomly selected genes, whose differential expression was detected by the SSH/microarray assays, in 18 pairs of HCC and the matched non-HCC liver tissues. We found the results were consistent with those obtained from SSH/microarray assays (Figure 6).

### Gene ontology analysis of the HCC differentially expressed genes

We compared the HCC up- and down-regulated transcriptomes identified by the SSH/microarray assays in accordance with their potential molecular functions, implicated biological processes and sub-cellular localization. As presented in Table 1, the HCC up-regulated transcriptomes had strong association with the regulation of cell cycle progression (19 vs 4, P = 0.052), transcription (44 vs. 13, P = 0.040), nucleic acid metabolism (80 vs. 26, P = 0.009), and protein metabolism (89 vs. 27, P = 0.002), while the HCC down-regulated transcriptomes tended to be related to loss of normal physiological function of the hepatocytes (complement and coagulation cascades, 0 vs. 11, P = 0.000), and impairment in cellular responses to extrinsic stresses including wounding (4 vs. 12, P = 0.011) and external biotic stimuli (10 vs. 15, P = 0.007), in cellular defense activities (16 vs. 24, P = 0.000), and in maintaining cell ion homeostasis (1 vs. 5, P = 0.021). In addition, genes related to cell responses to external growth stimuli and signal transduction (48 vs. 24, P > 0.05), and related to cell morphogenesis and biogenesis (28 vs. 9, P > 0.05) were more frequently found in the HCC up-regulated transcriptomes. On the other hand, the HCC up-regulated transcriptomes had more genes with their products distributed in cell nucleus (89 vs. 26, P = 0.001), while the HCC down-regulated transcriptomes had more genes of secreted proteins (4 vs. 15, P = 0.000).

**Table 1:** Gene ontology classification of the HCC differentially expressed genes

Up-regulated genes*	Down-regulated genes*	**P-value**	
Cell cycle	19	4	0.052
Regulation of apoptosis	8	1	0.121
Transcription & regulation of transcription	44	13	0.040
Signal transduction	48	24	0.777
Cell organizaiton & biogenesis	28	9	0.109
Purine & pyrimidine metabolism	80	26	0.009
Protein metabolism	89	27	0.002
Nuclear ^©^	89	26	0.001
Chromosome ^©^	8	3	0.432
Cytoskeleton ^©^	18	5	0.128
Ubiquitin ligase complex ^©^	7	1	0.170
Complement & coagulation cascades	0	11	0.000
Response to wounding	4	12	0.011
Defense response	16	24	0.000
Response to external biotic stimuli	10	15	0.007
Immune response	14	21	0.001
Cell ion homeostasis	1	5	0.021
Microsome ^©^	2	6	0.442
Peroxisome ^©^	1	4	0.051
Extra-cellular space ^©^	4	15	0.000

*Based on 360 and 202 informative up- and down-regulated genes identified using the SSH/microarray in HCC.

^© ^cellular components, based on 207 and 110 informative up- and down-regulated genes in human HCC, respectively.

P value was determined by Fisher's exact test.

## Discussion

Microarray techniques are limited in the detection of genes with low expression [14], while subtractive hybridization methods are restricted by their specificity and the tremendous work needed for validating the results, as well as for sequencing to identify genes. There have been many reports using microarray techniques for rapid and high throughput validation of the subtraction specificity of SSH by spotting the enriched clones on the chips or membranes [10-13,15-23]. However, such approaches would not only require tremendously sequencing works to identify genes including a great number of redundant clones in the subtracted amplicons, but also lose the sensitivity of SSH techniques to detect the low abundance transcripts, since the sensitivity of microarray analysis is determined by the targets used to hybridize chips but not by the probes printed on chips.

Herein we report our modified approaches. Instead of using the subtracted clones as probes spotted on chips, we directly labeled the enriched amplicons and used them as targets to hybridize the pre-made microarray chips for microarray analysis (named as "SSH/microarray" in this report). This approach allowed us not only to readily identify genes without tremendous sequencing works in the subtracted amplicons regardless of many redundant clones, but also to easily evaluate the subtraction efficiency and specificity. Moreover, the SSH/microarray approach made it possible to conduct a transcriptome-wide identification of differentially expressed genes particularly for those with low expression. It has been reported that the absolute expression level is not a crucial determinant for identifying genes, while the relative difference in expression levels does impact on whether or not a gene is recovered by subtractive hybridization [5,24]. In this report using the SSH/microarray approach, we identified about ten times more of the differentially expressed genes, particularly for those with low expression, in human hepatoma. Our findings successfully demonstrated the transcriptome-wide assays of differentially expressed genes at low abundance. This simple but very powerful approach would greatly facilitate future researches on functional genomics [15,19,25].

One of the main concerns about SSH techniques is their specificity. However, as combined with microarray techniques, the subtraction efficiency can be readily evaluated. As shown in Figure 2, the subtraction efficiency was determined by the rare presence of clones with equal hybridization intensities between the forward and reverse amplicons, and by the consistent exclusion of the house-keeping genes in the subtracted amplicons. The specificity was further confirmed by quantification of the differential gene expression between hepatoma and para-hepatoma liver tissues using RT real-time PCR in the 9 randomly selected genes, of which six (*GBA*, *PTPRF*, *GNL3*, *ERBB3*, *OGDH*, *FMO3*) were identified to be differentially expressed only by the SSH/microarray assays, but not the conventional cDNA microarrays assays

Of interest, using this novel approach, we identified many of the genes potentially implicated in hepatocellular carcinogenesis, which were not previously identified by conventional cDNA microarray assays. For examples, 18 genes (*WT1*, *NME2*, *IGF2*, *PPMIG*, *GMNN*, *PSMD2*, *CDK4*, *SFN*, *CDC20*, *CDC25C*, *AIF1*, *PINX1*, *CDC2*, *MSH2*, *PA2G4*, *ASPM*, *PAFAH1B1*, *DCTN3*) implicated in cell cycle regulation, 16 genes (*PLG*, *FABP3*, *APEG1*, *NCK1*, *RBBP4*, *NME2*, *CTBP1*, *FGA*, *IBFBP7*, *FGB*, *AIF1*, *CXCL1*, *JAG1*, *PINX1*, *GRN*, *PBEF1*) correlated with the regulation of cell proliferation, 16 genes (*HDAC1*, *DNASE1*, *NUDT2*, *CTNNAL1*, *HSAC1*, *BIRC1*, *FASTK*, *IGFBP3*, *TUBB*, *TNFSF10*, *TNFRSF11B*, *CLU*, *BFAR*, *BCAP31*, *BIRC2*, *C9*) involved in the pathways of cell death, 35 genes (*MYL4*, *APEG1*, *TTN*, *HTN1*, *MEST*, *EVL3*, *VLDLR*, *MYOG*, *TNNI2*, *HOXC11*, *IGF2*, *AHSG*, *TTID*, *BIRC1*, *PAFAH1B1*, *SYK*, *LMNA*, *TNFRSF11B*, *FALZ*, *BMP1*, *ETS2*, *NRD1*, *CXCL1*, *JAG1*, *SEMA4G*, *HEY1*, *ZNF22*, *CRMP1*, *TEAD4*, *IGFBP1*, *IGFBP3*, *IGFBP7*, *PTGS1*, *NEUGRIN*, *CUGBP1*) related to the regulation of cell differentiation and embryonic development, and 59 genes (*ASGR2*, *CAP2*, *RGS5*, *ITGB4BP*, *NCK1*, *EVL3*, *PLCG2*, *EPHA7*, *IGF2*, *IFNGR2*, *AHSG*, *CAP1*, *PTPRF*, *CXCL2*, *GNAI1*, *TGFBR1*, *NCSTN*, *DOK1*, *FRBB3*, *SYK*, *MC1R*, *CD79B*, *CSNKIE*, *CD69*, *AVPR1A*, *GNB2L1*, *ACVR1*, *BIRC2*, *FLT4*, *CXCL1*, *FAG1*, *PNOC*, *SLC9A3R1*, *LANCL1*, *GNB3*, *ADORA2B*, *FGR*, *NCK1*, *RAB11A*, *PLCG2*, *LOC91614*, *PRKAR1A*, *FLJ22595*, *ARF3*, *TYK2*, *MAP3K7IP1*, *MC1R*, *TNFSF10*, *ARF4*, *VAV2*, *AVPR1A*, *GNB2L1*, *MAPK10*, *STMN1*, *SNX17*, *ADORA2B*, *ECT2*, *RGS5*, *IGFBP3*) associated with signal transduction in response to extra-cellular proliferation and growth stimuli were found. Our findings that differentially expressed genes were related to multi-biological processes suggest the complexity of the molecular mechanisms for hepatocellular carcinogenesis.

## Conclusion

In this study, we modified the previously method of the combination of the suppressive subtractive hybridization and microarray techniques to differentially profile the low-expression transcriptomes of human hepatocellular carcinoma by directly labeling both of the reciprocal subtracted amplicons as target for cDNA microarray assays. Compared to SSH or previous SSH in conjunction with microarray approaches, this modified approach provided us with three additional advantages: 1) easy inspection of the subtraction efficiency, 2) avoidance of tremendous sequencing work for gene identification, 3) high sensitivity for identifying the low-expression, differentially expressed genes. This approach allowed for the detection of about ten times more of the differentially expressed genes than did the regular cDNA microarray approach in human hepatoma, particularly for those with low expression. Using this approach, we identified many genes potentially implicated in human hepatocarcinogenesis, which were not identified before. For its high efficiency and high sensitivity, this SSH/microarray approach is powerful for the rapid differentially profiling the low-expression transcriptomes, and most adequate for applying to functional genomic studies.

## Methods

### Tissues and patients

For SSH and cDNA microarray analysis, hepatoma and the corresponding non-cancerous liver tissues were obtained from 3 patients who had liver surgery at the Chang Gung Memorial Hospital. For reverse-transcription real-time PCR, hepatoma and the matched non-hepatoma liver tissues were obtained from additional 18 patients of hepatoma. Diagnoses of HCC and non-hepatoma liver tissues were based on histo-pathologic findings. The Internal Review Board for Medical Ethics of Chang Gung Memorial Hospital approved the specimen collection procedures and informed consent was obtained from each subject or subject's family.

### Suppressive subtractive hybridization

Total RNA from hepatoma and the para-hepatoma liver tissues and the poly(A) RNA was prepared as described before [26,27]. SSH was performed with the Clontech PCR-Select cDNA Subtraction Kit (Clontech Laboratories Inc., Palo Alto, CA) as described by the manufacturer but with the following modifications. Starting material consisted of 2 μg hepatoma mRNA as tester and 2 μg non-hepatoma liver tissue mRNA as driver, and vice versa. Primary and secondary PCR conditions were altered to increase specificity of amplification according to either plan A or B. Both A and B reduced the extension time and the number of cycles of the primary PCR to 2 min and 26 cycles, respectively. The primary PCR products were diluted 1/50 prior to use in the secondary PCR. All other aspects of plan A were as per the instructions of the manufacturer. Plan B diverged from plan A in two ways. First, the initial cycle of primary PCR was performed using annealing and extension times that had been reduced to 15 s and 1.5 min, respectively. Second, for subsequent cycles, the denaturing time was increased to 10 s while the annealing and extension times were reduced to 15 s and 1.5 min, respectively.

### RNA labeling and microarray procedures

In this study, we used the GMRCL Human 15 K set, Version 2 chips as previously described [28], which contained 14,811 sequence-verified, human cDNA clones mapped to 12,530 distinct genes. All of the samples for the regular cDNA microarray or SSH/microarray assays were performed with the dye-swapping microarray design for minimizing labeling bias and statistical variances of data. For the regular cDNA microarray experiment, we used 2 μg of the total RNA for labeling and hybridization using a 3DNA Array 350RP Detection kit (Genisphere, PA, USA).

For the SSH experiment, 1 μl subtractive PCR products were labeled with Cy3 and Cy *5*-dCTP (NEN, Boston, MA) using random primers. Unincorporated fluorescent nucleotides were removed using a Qiaquick PCR purification kit (Qiagen). The fluorescent-labeled DNAs were mixed with 30 μg of human cot-1 DNA (Invitrogen) and 100 μg yeast tRNA, precipitated and then resuspended in 30 μl of Microarray Hybridization Buffer Version 2 (Amersham Pharmacia). The hybridization solution was heated to 80°C for 10 min to denature the DNA and was then incubated for 30 min at 37°C, allowing cot-1DNA and yeast tRNA to block the repetitive sequences in genome probes. The probes were hybridized to a human cDNA microarray (GMRCL Human 15 K). We scanned the slides with a confocal scanner ChipReader (Virtek, Canada) and acquired the spot and background intensities with the GenePix Pro 4.1 software (Axon Instruments, Inc., CA, USA). The within-slide normalization was done using programs written with MATLAB 6.5 software (The MathWorks, Inc., MA, USA).

### Reverse-transcription real-time PCR

To validate the results obtained from the SSH/microarray assays, we randomly selected six differentially expressed genes identified only by the SSH/microarray assays and three differentially expressed genes identified by both the regular cDNA microarray and SSH/microarray assays for the comparison of gene expression between hepatoma and the corresponding non-hepatoma liver tissues in eighteen patients of HCC using reverse transcription real-time PCR (qRT-PCR). Total RNA was extracted from tissues with Trizol reagents and reverse transcribed using the SuperScript III first strand synthesis system (Invitrogen, Carlsbad, CA). qRT-PCR was conducted using the ABI PRISM 7000 sequence detection system (Applied Biosystems). Pre-designed Assays on Demand TaqMan probes and primer pairs for these 9 genes were obtained from Applied Biosystems Incoporated (ABI) (Foster City, CA). For each gene, two to four sets of Taq-Man probes and primers were tested. The probes contained a 6-carboxy-fluorescein phosphoramidite (FAM dye) label at the 5' end of the gene and a minor groove binder and non-fluorescent quencher at the 3' end. These were designed to hybridize across exon junctions. As a result, no fluorescent signal was generated by these assays when genomic DNA was used as a substrate, which confirmed that the assays measured only mRNA. Equal amounts of RNA were used for all qRT-PCR reactions, which were performed in triplicate, and 18S ribosomal RNAs were used as internal controls.

### Gene ontology analysis

All data (derived from three pairs of HCC and non-HCC liver tissues in a dye-swapping approach) were filtered so that 446 up-regulated and 255 down-regulated genes in HCC with at least two-fold difference and p value less than 0.01 were included in the further studies. After removal of the un-annotated genes, a total of 360 and 202 HCC up- and down-regulated genes were subjected to the subsequent gene ontology analyses. The two lists of the differentially expressed genes were analyzed using the on-line software FatiGo for comparative gene ontology categories including molecular function, biological process and cellular component [29]. They were also imported into the on-line software KEGG2 for pathway mapping [30,31]. The statistical significance was defined as P < 0.01 between the HCC up- and down-regulated gene groups using Fisher's exact test.

## Abbreviations

SSH, suppressive subtractive hybridization; HCC, hepatocellular carcinoma

## Authors' contributions

Y-S Pan and Y-L Lee conducted all of the experiments and contributed to data interpretation.

Y-S Lee performed data analysis.

W-C Lee collected all of the surgical samples.

S-Y Hsieh conceived and designed this study and was responsible for the manuscript preparation.

All authors read and approved the final manuscript.

## Supplementary Material

Additional File 1 **The list of the 69 and 701 differentially expressed genes**. A total of 69 and 701 differentially expressed genes identified via conventional cDNA microarray and the modified SSH/microarray approaches, respectively, are provided (Pan YS et al., 2006). Click here for file

## References

[B1] Schena M, Shalon D, Davis RW, Brown PO (1995). Quantitative monitoring of gene expression patterns with a complementary DNA microarray. Science.

[B2] Lockhart DJ, Dong H, Byrne C, Follettie MT, Gallo MV, Chee MS, Mittmann M, Wang C, Kobayashi M, Horton H, Brown EL (1996). Expression monitoring by hybridization to high-density oligonucleotide arrays. Nat Biotechnol.

[B3] Wen X, Fuhrman S, Michaels GS, Carr DB, Smith S, Barker JL, Somogyi R (1998). Large-scale temporal gene expression mapping of central nervous system development. Proc Natl Acad Sci USA.

[B4] Shyamsundar R, Kim YH, Higgins JP, Montgomery K, Jorden M, Sethuraman A, van de Rijn M, Botstein D, Brown PO, Pollack JR (2005). A DNA microarray survey of gene expression in normal human tissues. Genome Biol.

[B5] Diatchenko L, Lau YF, Campbell AP, Chenchik A, Moqadam F, Huang B, Lukyanov S, Lukyanov K, Gurskaya N, Sverdlov ED, Siebert PD (1996). Suppression subtractive hybridization: a method for generating differentially regulated or tissue-specific cDNA probes and libraries. Proc Natl Acad Sci USA.

[B6] Diatchenko L, Lukyanov S, Lau Y, Siebert P (1999). Suppression subtractive hybridization: a versatile method for identifying differentially expressed genes. Methods Enzymol.

[B7] Kuang WW, Thompson DA, Hoch RV, Weigel RJ (1998). Differential screening and suppression subtractive hybridization identified genes differentiallyexpressed in an estrogen receptor-positive breast carcinoma cell line. Nucleic Acids Res.

[B8] Harms C, Kase M, Hildebrandt A (2002). Characterization of minute differences between genomes of strains of Penicillium nalgiovense using subtractive suppression hybridization without cloning. Lett Appl Microbiol.

[B9] Rebrikov DV, Desai SM, Siebert PD, Lukyanov SA (2004). Suppression subtractive hybridization. Methods Mol Biol.

[B10] Yang GP, Ross DT, Kuang WW, Brown PO, Weigel RJ (1999). Combining SSH and cDNA microarrays for rapid identification of differentially expressed genes. Nucleic Acids Res.

[B11] Welford SM, Gregg J, Chen E, Garrison D, Sorensen PH, Denny CT, Nelson SF (1998). Detection of differentially expressed genes in primary tumor tissues using representational differences analysis coupled to microarray hybridization. Nucleic Acids Res.

[B12] Amatschek S, Koenig U, Auer H, Steinlein P, Pacher M, Gruenfelder A, Dekan G, Vogl S, Kubista E, Heider KH, Stratowa C, Schreiber M, Sommergruber W (2004). Tissue-wide expression profiling using cDNA subtraction and microarrays to identify tumor-specific genes. Cancer Res.

[B13] Bae JW, Rhee SK, Nam YD, Park YH (2005). Generation of subspecies level-specific microbial diagnostic microarrays using genes amplified from subtractive suppression hybridization as microarray probes. Nucleic Acids Res.

[B14] Rondeau G, McClelland M, Nguyen T, Risques R, Wang Y, Judex M, Cho AH, Welsh J (2005). Enhanced microarray performance using low complexity representations of the transcriptome. Nucleic Acids Res.

[B15] Rho J, Altmann CR., Socci ND, Merkov L, Kim N, So H, Lee O, Takami M, Brivanlou AH, Choi Y (2002). Gene expression profiling of osteoclast differentiation by combined suppression subtractive hybridization (SSH) and cDNA microarray analysis. DNA Cell Biol.

[B16] Malyala A, Pattee P, Nagalla SR, Kelly MJ, Ronnekleiv OK (2004). Suppression subtractive hybridization and microarrayidentification of estrogen-regulated hypothalamic genes. Neurochem Res.

[B17] Munir S, Singh S, Kaur K, Kapur V (2004). Suppression subtractive hybridization coupled with microarray analysis to examine differential expression of genes in virus infected cells. Biol Proced Online.

[B18] Hsieh SY, Chen WY, Shih TC, Yeh JY, Jeng JT (2005). Dys-regulation of clusterin in human hepatoma is not associated with tumorigenesis but is secondary to cell response to external tresses. Mol Carcinog.

[B19] Feng HC., Tsao SW, Ngan HY, Kwan HS, Shih SM, Xue WC, Chiu PM, Chan KW, Cheung AN (2005). Differential Gene Expression Identified in Complete Hydatidiform Mole by Combining Suppression Subtractive Hybridization and cDNA Microarray. Placenta.

[B20] Klebig C, Seitz S, Korsching E, Kristiansen G., Gustavus D, Scherneck S (2005). Profile of differentially expressedgenes after transfer of chromosome 17 into the breast cancer cell line CAL51. Genes Chromosomes Cancer.

[B21] Seo EY, Namkung JH, Lee KM, Lee WH, Im M, Kee SH, Tae Park G, Yang JM, Seo YJ, Park JK, Deok Kim C, Lee JH (2005). Analysis of calcium-inducible genes in keratinocytes using suppression subtractive hybridizationand cDNA microarray. Genomics.

[B22] Vallee M, Gravel C, Palin MF, Reghenas H, Stothard P, Wishart DS, Sirard MA (2005). Identification of novel and known oocyte-specificgenes using complementary DNA subtraction and microarray analysis inthree different species. Biol Reprod.

[B23] Ghorbel MT, Sharman G, Hindmarch C, Becker KG, Barrett T, Murphy D (2006). Microarray screening of suppression subtractive hybridization-PCR cDNA libraries identifies novel RNAs regulated by dehydration in the rat supraoptic nucleus. Physiol Genomics.

[B24] Braun BS, Frieden R, Lessnick SL, May WA, Denny CT (1995). Identification of target genes for the Ewing's sarcoma EWS/FLI fusion protein by representational difference analysis. Mol Cell Biol.

[B25] Seta KA, Millhorn DE (2004). Functional genomics approach to hypoxia signaling. J Appl Physiol.

[B26] Hsieh SY, Yang PY, Ou JT, Chu CM, Liaw YF (1994). Polyadenylation of the mRNA of hepatitis delta virus is dependent on the structure of the nascent RNA and regulated by the small or large delta antigen. Nucleic Acids Res.

[B27] Hsieh SY, Liaw SF, Lee SN, Hsieh PS, Lin KH, Chu CM, Liaw YF (2003). Aberrant Caspase-activated DNase (CAD) gene in human hepatoma cells. Br J Cancer.

[B28] Lee YS, Chen CH, Chao A, Chen ES, Wei ML, Chen LK, Yang KD, Lin MC, Wang YH, Liu JW, Eng HL, Chiang PC, Wu TS, Tsao KC, Huang CG, Tien YJ, Wang TH, Wang HS, Lee YS (2005). Molecular signature of clinical severity in recovering patients with severe acute respiratory syndrome coronavirus (SARS-CoV). BMC Genomics.

[B29] FatiGO: a web tool for finding significant associations of Gene Ontology terms with groups of genes. http://fatigo.bioinfo.cipf.es/.

[B30] Bono H, Ogata H, Goto S, Kanehisa M (1998). Reconstruction of amino acid biosynthesis pathways from the complete genome sequence. Genome Res.

[B31] KEGG 2: Kyoto Encyclopedia of Genes and Genomes. http://www.genome.ad.jp/kegg/.

