# Supplementary material for: Differentially profiling the low-expression transcriptomes of human hepatoma using a novel SSH/microarray approach
Source: BMC Genomics. 2006 May 31;7:131. doi: 10.1186/1471-2164-7-131 (PMC1522022; doi:10.1186/1471-2164-7-131)
Supplement: Additional File 1 — The list of the 69 and 701 differentially expressed genes. A total of 69 and 701 differentially expressed genes identified via conventional cDNA microarray and the modified SSH/microarray approaches, respectively, are provided (Pan YS et al., 2006). [file 1471-2164-7-131-S1.pdf]

TableS 1a :43 De-regulate at SSH and cDNA base on average fold change > 2 or < 0.5 and p value < 0.01 (t te

Gene Name	Symbol	UniGene ID	Cytoband	P value in SSH	P value in cDNA
complement component 9	C9	Hs.1290	5p14-p12	0.0006	0.0010
tryptophan 2,3-dioxygenase	TDO2	Hs.183671	4q31-q32	0.0000	0.0029
REV3-like, catalytic subunit of DNA polymerase zeta	REV3L	Hs.232021	6q21	0.0020	0.0087
interleukin 1 receptor-like 1	IL1RL1	Hs.66	2q12	0.0025	0.0039
UDP-glucose pyrophosphorylase 2	UGP2	Hs.417361	2p14-p13	0.0001	0.0003
protein phosphatase 1, regulatory (inhibitor) subunit 3	PPP1R3C	Hs.303090	10q23-q24	0.0000	0.0003
metallothionein 1F (functional)	MT1F	Hs.438737	16q13	0.0002	0.0072
tubulin, alpha, ubiquitous	K-ALPHA-1	Hs.446608	12q13.12	0.0059	0.0000
hypothetical protein FLJ33641	FLJ33641	Hs.44930	5q11.2	0.0055	0.0018
CDNA FLJ12815 fis, clone NT2RP2002546	EST	Hs.27621		0.0029	0.0002
Similar to 40S ribosomal protein SA (P40) (34/67 kDa	EST	Hs.448968	19p12	0.0001	0.0015
fetal Alzheimer antigen	FALZ	Hs.440947	17q24.3	0.0074	0.0018
alpha-2-HS-glycoprotein	AHSG	Hs.324746	3q27	0.0047	0.0068
UDP glycosyltransferase 1 family, polypeptide A10	UGT1A10	Hs.278896		0.0004	0.0003
4-hydroxyphenylpyruvate dioxygenase	HPD	Hs.2899	12q24-qter	0.0029	0.0001
catenin (cadherin-associated protein), alpha-like 1	CTNNAL1	Hs.58488	9q31.2	0.0044	0.0058
COX15 homolog, cytochrome c oxidase assembly prot	COX15	Hs.226581	10q24	0.0001	0.0020
thiopurine S-methyltransferase	TPMT	Hs.377402	6p22.3	0.0021	0.0001
lipoyltransferase 1	LIPT1	Hs.112356	2q11.2	0.0010	0.0000
fatty acid binding protein 3, muscle and heart (mamma	FABP3	Hs.112669	1p33-p32	0.0019	0.0020
tryptophan 2,3-dioxygenase	TDO2	Hs.183671	4q31-q32	0.0003	0.0007
villin 2 (ezrin)	VIL2	Hs.403997	6q25.2-q26	0.0024	0.0000
tubulin, alpha, ubiquitous	K-ALPHA-1	Hs.446608	12q13.12	0.0039	0.0041
4-hydroxyphenylpyruvate dioxygenase	HPD	Hs.2899	12q24-qter	0.0000	0.0055
complement component 9	C9	Hs.1290	5p14-p12	0.0003	0.0000
metallothionein 1F (functional)	MT1F	Hs.438737	16q13	0.0000	0.0001
hypothetical protein FLJ23235	FLJ23235	Hs.211501	4p14	0.0000	0.0075
zinc finger protein 519	ZNF519	Hs.352635	18p11.21	0.0000	0.0007
solute carrier family 38, member 2	SLC38A2	Hs.298275	12q	0.0010	0.0031
metallothionein 1A (functional)	MT1A	Hs.440939	16q13	0.0000	0.0001
hypothetical protein LOC51244	LOC51244	Hs.236257	3p25.1	0.0060	0.0009
chemokine (C-X-C motif) ligand 2	CXCL2	Hs.75765	4q21	0.0062	0.0009
golgi reassembly stacking protein 2, 55kDa	GORASP2	Hs.6880	2q31.1-q31.2	0.0096	0.0049
Transcribed sequence with moderate similarity to prot	EST	Hs.447471		0.0062	0.0061
metallothionein 2A	MT2A	Hs.418241	16q13	0.0000	0.0015
chromosome 20 open reading frame 64	C20orf64	Hs.440263	20q13.2	0.0003	0.0035
hypothetical protein FLJ20445	FLJ20445	Hs.343748	10q23.32-q23.3	0.0070	0.0088
insulin-like growth factor binding protein 1	IGFBP1	Hs.401316	7p13-p12	0.0002	0.0070
metallothionein 1X	MT1X	Hs.374950	16q13	0.0001	0.0008
metallothionein 1K	MT1K	Hs.188518	16q13	0.0000	0.0079
PR domain containing 10	PRDM10	Hs.275086	11q25	0.0046	0.0003
tubulin, alpha, ubiquitous	K-ALPHA-1	Hs.446608	12q13.12	0.0033	0.0000
stathmin 1/oncoprotein 18	STMN1	Hs.209983	1p36.1-p35	0.0090	0.0034

TableS 1b : 69 De-regulate at cDNA base on average fold change > 2 or < 0.5 and p value < 0.01 (T test)					
				P value in	
Gene Name	Symbol	UniGene ID	Cytoband	SSH	cDNA
translational inhibitor protein p14.5	UK114	Hs.18426	8q22	0.0133	0.0081
complement component 9	C9	Hs.1290	5p14-p12	0.0006	0.0010
histone 1, H4j	HIST1H4J	Hs.278483	6p22-p21.3	0.0272	0.0037
paternally expressed 3	PEG3	Hs.201776		0.0113	0.0026
hemopexin	HPX	Hs.426485	11p15.5-p15.4	0.0117	0.0009
tryptophan 2,3-dioxygenase	TDO2	Hs.183671	4q31-q32	0.0000	0.0029
REV3-like, catalytic subunit of DNA polymerase zeta	REV3L	Hs.232021	6q21	0.0020	0.0087
interleukin 1 receptor-like 1	IL1RL1	Hs.66	2q12	0.0025	0.0039
UDP-glucose pyrophosphorylase 2	UGP2	Hs.417361	2p14-p13	0.0001	0.0003
protein phosphatase 1, regulatory (inhibitor) subunit 3	PPP1R3C	Hs.303090	10q23-q24	0.0000	0.0003
metallothionein 1F (functional)	MT1F	Hs.438737	16q13	0.0002	0.0072
deformed epidermal autoregulatory factor 1 (Drosophi	DEAF1	Hs.428112	11p15.5	0.0108	0.0005
tubulin, alpha, ubiquitous	K-ALPHA-1	Hs.446608	12q13.12	0.0059	0.0000
hypothetical protein FLJ33641	FLJ33641	Hs.44930	5q11.2	0.0055	0.0018
solute carrier family 39 (zinc transporter), member 14	SLC39A14	Hs.301743	8p21.3	0.0173	0.0016
CDNA FLJ12815 fis, clone NT2RP2002546	EST	Hs.27621		0.0029	0.0002
Transcribed sequences	EST	Hs.459670		0.0414	0.0049
MRNA; cDNA DKFZp313I1032 (from clone DKFZp3	EST	Hs.321261		0.8839	0.0049
dermatan sulfate proteoglycan 3	DSPG3	Hs.435680	12q21	0.0765	0.0026
Similar to 40S ribosomal protein SA (P40) (34/67 kDa	EST	Hs.448968	19p12	0.0001	0.0015
fetal Alzheimer antigen	FALZ	Hs.440947	17q24.3	0.0074	0.0018
alpha-2-HS-glycoprotein	AHSG	Hs.324746	3q27	0.0047	0.0068
pleiomorphic adenoma gene-like 1	PLAGL1	Hs.132911		0.1459	0.0003
serine (or cysteine) proteinase inhibitor, clade A (alp	SERPINA3	Hs.76353	14q32.1	0.0323	0.0013
jumonji domain containing 2A	JMJD2A	Hs.155983	1p34.2-p34.1	0.0291	0.0036
UDP glycosyltransferase 1 family, polypeptide A10	UGT1A10	Hs.278896		0.0004	0.0003
4-hydroxyphenylpyruvate dioxygenase	HPD	Hs.2899	12q24-qter	0.0029	0.0001
catenin (cadherin-associated protein), alpha-like 1	CTNNAL1	Hs.58488	9q31.2	0.0044	0.0058
COX15 homolog, cytochrome c oxidase assembly prot	COX15	Hs.226581	10q24	0.0001	0.0020
polymerase (RNA) II (DNA directed) polypeptide I, 14	POLR2I	Hs.47062	19q12	0.0171	0.0022
mannosyl (alpha-1,6-)-glycoprotein beta-1,2-N-acetylg	MGAT2	Hs.93338	14q21	0.2976	0.0043
thiopurine S-methyltransferase	TPMT	Hs.377402	6p22.3	0.0021	0.0001
lipoyltransferase 1	LIPT1	Hs.112356	2q11.2	0.0010	0.0000
fatty acid binding protein 3, muscle and heart (mamma	FABP3	Hs.112669	1p33-p32	0.0019	0.0020
tryptophan 2,3-dioxygenase	TDO2	Hs.183671	4q31-q32	0.0003	0.0007
villin 2 (ezrin)	VIL2	Hs.403997	6q25.2-q26	0.0024	0.0000
tubulin, alpha, ubiquitous	K-ALPHA-1	Hs.446608	12q13.12	0.0039	0.0041
tubulin, alpha, ubiquitous	K-ALPHA-1	Hs.446608	12q13.12	0.0184	0.0050
CD48 antigen (B-cell membrane protein)	CD48	Hs.901	1q21.3-q22	0.0302	0.0089
4-hydroxyphenylpyruvate dioxygenase	HPD	Hs.2899	12q24-qter	0.0000	0.0055
complement component 9	C9	Hs.1290	5p14-p12	0.0003	0.0000
metallothionein 1F (functional)	MT1F	Hs.438737	16q13	0.0000	0.0001
hypothetical protein FLJ23235	FLJ23235	Hs.211501	4p14	0.0000	0.0075
zinc finger protein 519	ZNF519	Hs.352635	18p11.21	0.0000	0.0007
solute carrier family 38, member 2	SLC38A2	Hs.298275	12q	0.0010	0.0031
metallothionein 1A (functional)	MT1A	Hs.440939	16q13	0.0000	0.0001
hypothetical protein LOC51244	LOC51244	Hs.236257	3p25.1	0.0060	0.0009
chemokine (C-X-C motif) ligand 2	CXCL2	Hs.75765	4q21	0.0062	0.0009
golgi reassembly stacking protein 2, 55kDa	GORASP2	Hs.6880	2q31.1-q31.2	0.0096	0.0049
Transcribed sequence with moderate similarity to prote	EST	Hs.447471		0.0062	0.0061
hemoglobin, alpha 2	HBA2	Hs.449630	16p13.3	0.1836	0.0023
CDNA clone MGC:52263 IMAGE:4123447, complete	EST	Hs.251664		0.0268	0.0011
Transcribed sequence with moderate similarity to prote	EST	Hs.447471		0.0184	0.0000
Clone IMAGE:4824631, mRNA	EST	Hs.385640		0.0065	0.0057
Clone IMAGE:5272798, mRNA	EST	Hs.131463		0.0817	0.0011
Similar to Nonhistone chromosomal protein HMG-14	EST	Hs.469373	18q21.31	0.0396	0.0002
metallothionein 2A	MT2A	Hs.418241	16q13	0.0000	0.0015
chromosome 20 open reading frame 64	C20orf64	Hs.440263	20q13.2	0.0003	0.0035

hypothetical protein FLJ20445	FLJ20445	Hs.343748	10q23.32-q23.3	0.0070	0.0088
hypothetical protein BC011204	LOC113828	Hs.197680	22q13.1	0.0124	0.0082
mitogen-activated protein kinase 6	MAPK6	Hs.271980	15q21	0.0146	0.0050
insulin-like growth factor binding protein 1	IGFBP1	Hs.401316	7p13-p12	0.0002	0.0070
KIAA1173 protein	KIAA1173	Hs.27566	3p22.1	0.2857	0.0025
metallothionein 1X	MT1X	Hs.374950	16q13	0.0001	0.0008
metallothionein 1K	MT1K	Hs.188518	16q13	0.0000	0.0079
PR domain containing 10	PRDM10	Hs.275086	11q25	0.0046	0.0003
Similar to tubulin, beta 5 (LOC92755), mRNA	EST	Hs.458472	8p12	0.0330	0.0075
tubulin, alpha, ubiquitous	K-ALPHA-1	Hs.446608	12q13.12	0.0033	0.0000
stathmin 1/oncoprotein 18	STMN1	Hs.209983	1p36.1-p35	0.0090	0.0034

TableS 1c : 701 De-regulate at SSH base on average fold change > 2 or < 0.5 and p value < 0.01 (t test)

Gene Name	Symbol	UniGene ID	Cytoband	P value in SSH	P value in cDNA
Transcribed sequence with strong similarity to protein	EST	Hs.485572		0.0002	0.0249
complement component 9	C9	Hs.1290	5p14-p12	0.0006	0.0010
arginase, liver	ARG1	Hs.440934	6q23	0.0013	0.0140
high mobility group nucleosomal binding domain 4	HMGN4	Hs.236774	6p21.3	0.0000	0.2672
general transcription factor IIA, 2, 12kDa	GTF2A2	Hs.279528	15q22.2	0.0019	0.2512
haptoglobin	HP	Hs.403931	16q22.1	0.0050	0.1036
chromosome 18 open reading frame 1	C18orf1	Hs.285091	18p11.2	0.0055	0.0718
ribosomal protein L37a	RPL37A	Hs.433701	2q35	0.0055	0.0584
tubulin alpha 6	TUBA6	Hs.406578	12q12-q14	0.0021	0.0574
hydroxysteroid (17-beta) dehydrogenase 4	HSD17B4	Hs.356894	5q21	0.0000	0.9241
chymotrypsin C (caldecrin)	CTRC	Hs.8709	1p36.21	0.0061	1.0000
tumor necrosis factor receptor superfamily, member 1	TNFRSF11B	Hs.81791	8q24	0.0050	0.0249
pancreatic lipase-related protein 1	PNLIPRP1	Hs.73923	10q25.3	0.0030	0.8543
v-ets erythroblastosis virus E26 oncogene homolog 2 (	ETS2	Hs.292477	21q22.3	0.0000	0.0404
RAB11A, member RAS oncogene family	RAB11A	Hs.75618	15q21.3-q22.31	0.0084	0.3632
allograft inflammatory factor 1	AIF1	Hs.76364	6p21.3	0.0083	0.1384
cathepsin S	CTSS	Hs.181301	1q21	0.0083	0.1859
tryptophan 2,3-dioxygenase	TDO2	Hs.183671	4q31-q32	0.0000	0.0029
hemoglobin, beta	HBB	Hs.155376	11p15.5	0.0092	0.0360
gamma-glutamyl hydrolase (conjugase, folylpolygamn	GGH	Hs.78619	8q12.3	0.0033	0.1567
haptoglobin	HP	Hs.403931	16q22.1	0.0017	0.0141
malate dehydrogenase 1, NAD (soluble)	MDH1	Hs.75375	2p13.3	0.0001	0.1841
REV3-like, catalytic subunit of DNA polymerase zeta	REV3L	Hs.232021	6q21	0.0020	0.0087
CD69 antigen (p60, early T-cell activation antigen)	CD69	Hs.82401	12p13-p12	0.0032	0.1757
interleukin 1 receptor-like 1	IL1RL1	Hs.66	2q12	0.0025	0.0039
fibrinogen-like 1	FGL1	Hs.107	8p22-p21.3	0.0015	0.0158
LanC lantibiotic synthetase component C-like 1 (bacte	LANCL1	Hs.13351	2q33-q35	0.0019	0.1221
MRNA, chromosome 1 specific transcript KIAA0507.	EST	Hs.497770		0.0092	0.3632
solute carrier family 33 (acetyl-CoA transporter), mem	SLC33A1	Hs.285176	3q25.31	0.0021	0.0145
titin	TTN	Hs.434384		0.0017	0.0308
2,4-dienoyl CoA reductase 1, mitochondrial	DECR1	Hs.414754	8q21.3	0.0004	0.0495
chromosome 14 open reading frame 45	C14orf45	Hs.260555	14q24.3	0.0063	0.2029
hypothetical protein MGC10854	MGC10854	Hs.410924	12q24.11	0.0000	0.0240
major histocompatibility complex, class II, DR beta 3	HLA-DRB3	Hs.308026		0.0039	0.0645
translin-associated factor X	TSNAX	Hs.96247	1q42.1	0.0042	0.0593
UDP-glucose pyrophosphorylase 2	UGP2	Hs.417361	2p14-p13	0.0001	0.0003
heat shock transcription factor 2	HSF2	Hs.158195	6q22.31	0.0060	0.1691
hepsin (transmembrane protease, serine 1)	HPN	Hs.432750	19q11-q13.2	0.0006	0.6065
Transcribed sequences	EST	Hs.37138		0.0027	0.0489
solute carrier family 26, member 3	SLC26A3	Hs.1650	7q31	0.0028	0.4946
upstream binding transcription factor, RNA polymeras	UBTF	Hs.89781	17q21.3	0.0063	0.3972
sialyltransferase 7 ((alpha-N-acetylneuraminyl-2,3-bet	SIAT7B	Hs.288215	17q25.1	0.0006	0.0411
protein phosphatase 1, regulatory (inhibitor) subunit 3	PPP1R3C	Hs.303090	10q23-q24	0.0000	0.0003
metallothionein 1F (functional)	MT1F	Hs.438737	16q13	0.0002	0.0072
guanine nucleotide binding protein (G protein), alpha i	GNAI1	Hs.203862	7q21	0.0004	0.8412
carbonic anhydrase III, muscle specific	CA3	Hs.82129	8q13-q22	0.0006	0.3632
B-cell receptor-associated protein 31	BCAP31	Hs.381232	Xq28	0.0062	0.1813
spermidine/spermine N1-acetyltransferase	SAT	Hs.28491	Xp22.1	0.0004	0.0774
capping protein (actin filament) muscle Z-line, alpha 2	CAPZA2	Hs.369579	7q31.2-q31.3	0.0083	0.8124
enolase 3, (beta, muscle)	ENO3	Hs.224171	17pter-p11	0.0001	0.2470
ribonuclease, RNase A family, 4	RNASE4	Hs.283749		0.0060	0.1237
Data not found	EST	Hs.446682		0.0028	0.1075
actin related protein 2/3 complex, subunit 3, 21kDa	ARPC3	Hs.439511	12q24.11	0.0007	0.3499
protein phosphatase 1G (formerly 2C), magnesium-de	PPM1G	Hs.17883	2p23.3	0.0075	0.2618
methionine-tRNA synthetase	MARS	Hs.355867	12q13.2	0.0011	0.1070
enhancer of zeste homolog 2 (Drosophila)	EZH2	Hs.444082	7q35-q36	0.0068	0.1898
stratifin	SFN	Hs.184510	1p36.11	0.0065	0.0221
forkhead box M1	FOXM1	Hs.479302	12p13	0.0085	0.3632

retinoblastoma binding protein 7	RBBP7	Hs.406078	Xp22.2	0.0009	0.0313
hypothetical protein MGC5178	MGC5178	Hs.458369	16p11.2	0.0001	0.0560
sortilin 1	SORT1	Hs.394609	1p21.3-p13.1	0.0047	0.2104
zinc finger protein 211	ZNF211	Hs.449970	19q13.4	0.0038	0.1334
ribosomal protein L18a	RPL18A	Hs.337766	19p13	0.0072	0.0137
polymerase (RNA) III (DNA directed) (62kD)	RPC62	Hs.250745	1q21.1	0.0089	0.1880
prion protein interacting protein	PRNPIP	Hs.151032	1p32	0.0057	0.1869
ubiquitin fusion degradation 1-like	UFD1L	Hs.404525	22q11.21	0.0008	0.1021
N-ethylmaleimide-sensitive factor attachment protein,	NAPA	Hs.75932	19q13.32	0.0006	0.0597
MCM7 minichromosome maintenance deficient 7 (S. c	MCM7	Hs.438720	7q21.3-q22.1	0.0078	0.0297
CUG triplet repeat, RNA binding protein 1	CUGBP1	Hs.321390	11p11	0.0012	0.2712
ring finger protein (C3HC4 type) 8	RNF8	Hs.24439	6p21.3	0.0075	0.1217
KIAA0296 gene product	KIAA0296	Hs.458489	16p11.2	0.0022	0.2822
cAMP responsive element binding protein-like 1	CREBL1	Hs.42853		0.0058	0.0831
H2A histone family, member V	H2AV	Hs.301005	7p13	0.0072	0.0009
spleen tyrosine kinase	SYK	Hs.192182	9q22	0.0000	0.0526
eukaryotic translation initiation factor 4A, isoform 1	EIF4A1	Hs.129673	17p13	0.0028	0.1147
Transcribed sequence with strong similarity to protein	EST	Hs.478508		0.0011	0.9941
prostaglandin-endoperoxide synthase 1 (prostaglandin	PTGS1	Hs.88474	9q32-q33.3	0.0057	0.0506
hypothetical protein LOC153561	LOC153561	Hs.512407	5q13.2	0.0001	0.0993
mitogen-activated protein kinase kinase kinase 7 intera	MAP3K7IP1	Hs.403927	22q13.1	0.0057	0.0016
tubulin, alpha, ubiquitous	K-ALPHA-1	Hs.446608	12q13.12	0.0059	0.0000
phosphatidylserine receptor	PTDSR	Hs.72660	17q25	0.0059	0.3632
chemokine (C-X-C motif) ligand 1 (melanoma growth	CXCL1	Hs.789	4q21	0.0000	0.7466
proteasome (prosome, macropain) 26S subunit, non-A'	PSMD2	Hs.388921	3q27.1	0.0001	0.5194
barrier to autointegration factor 1	BANF1	Hs.433759	11q13.1	0.0096	0.0859
nicastrin	NCSTN	Hs.4788	1q22-q23	0.0090	0.0799
RNA binding motif protein 6	RBM6	Hs.188879	3p21.3	0.0038	0.3632
CAP, adenylate cyclase-associated protein, 2 (yeast)	CAP2	Hs.296341	6p22.3	0.0038	0.2790
prepronociceptin	PNOC	Hs.371809	8p21	0.0008	0.0619
docking protein 1, 62kDa (downstream of tyrosine kin	DOK1	Hs.103854	2p13	0.0077	0.4240
Clone CB1A11 immunoglobulin heavy chain variable	EST	Hs.448830		0.0017	0.0923
major histocompatibility complex, class II, DR beta 3	HLA-DRB3	Hs.308026		0.0032	0.0363
manic fringe homolog (Drosophila)	MFNG	Hs.371768	22q12	0.0064	0.1492
dishevelled, dsh homolog 3 (Drosophila)	DVL3	Hs.381928	3q27	0.0007	0.3319
major histocompatibility complex, class II, DP alpha 1	HLA-DPA1	Hs.914	6p21.3	0.0012	0.0169
major histocompatibility complex, class II, DP beta 1	HLA-DPB1	Hs.368409	6p21.3	0.0009	0.0144
CD72 antigen	CD72	Hs.116481	9p13.3	0.0016	0.6825
zinc finger and BTB domain containing 5	ZBTB5	Hs.3682	9p13.2	0.0015	0.0526
KIAA0746 protein	KIAA0746	Hs.49500	4p15.2	0.0063	0.4355
SWI/SNF related, matrix associated, actin dependent r	SMARCD3	Hs.444445	7q35-q36	0.0025	0.0562
nitrogen fixation cluster-like	NIFU	Hs.350702	12q24.1	0.0036	0.0105
solute carrier family 25 (mitochondrial carrier; phosph	SLC25A3	Hs.290404	12q23	0.0000	0.0084
sorting nexin 17	SNX17	Hs.278569	2p23-p22	0.0042	0.0090
guanine nucleotide binding protein (G protein), beta po	GNB3	Hs.3759	12p13	0.0088	0.0078
Transcribed sequence with moderate similarity to prote	EST	Hs.447471		0.0023	0.0145
CD79B antigen (immunoglobulin-associated beta)	CD79B	Hs.89575	17q23	0.0081	0.0512
dynactin 3 (p22)	DCTN3	Hs.511768	9p13	0.0006	0.1301
amyotrophic lateral sclerosis 2 (juvenile) chromosome	ALS2CR3	Hs.154248	2q33	0.0078	0.0449
baculoviral IAP repeat-containing 2	BIRC2	Hs.289107	11q22	0.0093	0.2858
solute carrier family 10 (sodium/bile acid cotransporte	SLC10A1	Hs.952	14q24.1	0.0019	0.0977
betaine-homocysteine methyltransferase	BHMT	Hs.80756	5q13.1-q15	0.0004	0.0195
Transcribed sequences	EST	Hs.271280		0.0020	0.1242
hypothetical protein FLJ33641	FLJ33641	Hs.44930	5q11.2	0.0055	0.0018
visinin-like 1	VSNL1	Hs.2288	2p24.3	0.0099	0.4459
actin related protein 2/3 complex, subunit 3, 21kDa	ARPC3	Hs.439511	12q24.11	0.0007	0.1536
inhibitor of DNA binding 2, dominant negative helix-l	ID2	Hs.180919	2p25	0.0001	0.0160
tankyrase 1 binding protein 1, 182kDa	TNKS1BP1	Hs.9645	11q12.1	0.0011	0.3632
pyridoxine-5'-phosphate oxidase	PNPO	Hs.327335	17q21.32	0.0007	0.0763
solute carrier family 6 (neurotransmitter transporter,	SLC6A1	Hs.22003	3p25-p24	0.0085	0.1950
protein kinase, AMP-activated, gamma 1 non-catalytic	PRKAG1	Hs.3136	12q12-q14	0.0010	0.9741

syndecan 3 (N-syndecan)	SDC3	Hs.158287	1pter-p22.3	0.0055	0.1496
Rho guanine nucleotide exchange factor (GEF) 5	ARHGEF5	Hs.334	7q33-q35	0.0080	0.9139
estrogen-related receptor gamma	ESRRG	Hs.151017	1q41	0.0016	0.3632
KIAA0635	KIAA0635	Hs.185091	4q12	0.0020	0.2419
hypothetical protein FLJ10597	FLJ10597	Hs.90375	1p34.1	0.0078	0.3632
CDNA FLJ12815 fis, clone NT2RP2002546	EST	Hs.27621		0.0029	0.0002
iduronidase, alpha-L-	IDUA	Hs.89560	4p16.3	0.0017	0.0929
cathepsin W (lymphopain)	CTSW	Hs.416848	11q13.1	0.0000	0.0520
hypothetical protein LOC283578	LOC283578	Hs.212992	14q24.3	0.0080	0.0989
HLA-B associated transcript 1	BAT1	Hs.254042	6p21.3	0.0096	0.7549
cytochrome P450, family 2, subfamily A, polypeptide	CYP2A13	Hs.181973	19q13.2	0.0007	0.0209
PTPRF interacting protein, binding protein 1 (liprin be	PPFIBP1	Hs.389023	12p11.23-p11.2	0.0001	0.1088
insulin-like growth factor binding protein 7	IGFBP7	Hs.435795	4q12	0.0044	0.4481
ankyrin repeat domain 15	ANKRD15	Hs.77546	9p24.3	0.0025	0.2285
ribonuclease, RNase A family, 4	RNASE4	Hs.283749		0.0033	0.1162
pericentrin 1	PCNT1	Hs.184352	17q25.1	0.0059	0.3632
chromosome 6 open reading frame 133	C6orf133	Hs.131842	6p21.1	0.0090	0.3632
ret finger protein	RFP	Hs.440382	6p22	0.0055	0.3632
YY1 transcription factor	YY1	Hs.388927	14q	0.0008	0.1766
mitochondrial ribosomal protein L9	MRPL9	Hs.288936		0.0007	0.3632
calcium channel, voltage-dependent, gamma subunit 2	CACNG2	Hs.268545	22q13.1	0.0010	0.3632
cullin 4B	CUL4B	Hs.155976	Xq23	0.0022	0.9485
acid cluster protein 33	ACP33	Hs.242458	15q21-q22	0.0042	0.7496
non-metastatic cells 2, protein (NM23B) expressed in	NME2	Hs.433416	17q21.3	0.0097	0.1031
putative membrane protein	LOC54499	Hs.93832	1q22-q25	0.0014	0.0569
platelet-activating factor acetylhydrolase, isoform Ib,	PAFAH1B1	Hs.77318	17p13.3	0.0001	1.0000
FAST kinase	FASTK	Hs.75087	7q35	0.0041	0.2716
myo-inositol 1-phosphate synthase A1	ISYNA1	Hs.405873	19p13.11	0.0004	0.0778
chromosome 1 open reading frame 19	C1orf19	Hs.440663	1q25	0.0070	1.0000
phosphoribosylaminoimidazole carboxylase, phosphor	PAICS	Hs.444439	4pter-q21	0.0063	1.0000
acidic (leucine-rich) nuclear phosphoprotein 32 family	ANP32B	Hs.459987	9q22.32	0.0015	0.1318
single-stranded DNA binding protein 1	SSBP1	Hs.923	7q34	0.0076	0.4752
phospholipase A2, group IIA (platelets, synovial fluid)	PLA2G2A	Hs.76422	1p35	0.0001	0.1002
metastais-associated gene family, member 2	MTA2	Hs.173043	11q12-q13.1	0.0026	0.3632
F-box and leucine-rich repeat protein 5	FBXL5	Hs.5548	4p15.33	0.0014	0.1689
adenosine deaminase, RNA-specific	ADAR	Hs.7957	1q21.1-q21.2	0.0019	0.0463
ATP synthase, H+ transporting, mitochondrial F0 com	ATP5G2	Hs.89399	12q13.13	0.0006	0.0587
hypothetical protein MGC3047	MGC3047	Hs.76239	1p36.33	0.0012	0.6875
ATPase, H+ transporting, lysosomal accessory protein	ATP6AP2	Hs.183434	Xq21	0.0043	0.4121
CDNA FLJ11381 fis, clone HEMBA1000501	EST	Hs.127797		0.0004	0.0168
cytochrome P450, family 3, subfamily A, polypeptide	CYP3A7	Hs.308638	7q21-q22.1	0.0013	0.3138
hypothetical protein FLJ22625	FLJ22625	Hs.106534	5q31.1	0.0066	0.2090
kaptin (actin binding protein)	KPTN	Hs.25441	19q13.32	0.0014	0.1973
geranylgeranyl diphosphate synthase 1	GGPS1	Hs.55498	1q43	0.0031	0.9557
CDNA FLJ37123 fis, clone BRACE2022450	EST	Hs.337266		0.0014	0.3632
syndecan 2 (heparan sulfate proteoglycan 1, cell surfac	SDC2	Hs.1501	8q22-q23	0.0019	0.0667
bifunctional phosphopantetheine adenylyl transferase/c	DPCK	Hs.296422	17q12-q21	0.0016	0.5192
AD24 protein	AD24	Hs.74899	10q23.33	0.0008	0.3632
histone deacetylase 6	HDAC6	Hs.6764	Xp11.23	0.0033	0.3632
melanoma antigen, family A, 10	MAGEA10	Hs.18048	Xq28	0.0073	1.0000
methylmalonyl Coenzyme A mutase	MUT	Hs.155212	6p21	0.0000	0.0356
kallikrein B, plasma (Fletcher factor) 1	KLKB1	Hs.1901	4q34-q35	0.0001	0.0484
MAWD binding protein	MAWBP	Hs.16341	10pter-q25.3	0.0004	0.0342
COMM domain containing 10	COMMD10	Hs.151458	5q23.1	0.0006	0.0649
complement component 1, s subcomponent	C1S	Hs.458355	12p13	0.0098	0.0012
Transcribed sequences	EST	Hs.35125		0.0083	0.0935
fucosyltransferase 1 (galactoside 2-alpha-L-fucosyltran	FUT1	Hs.69747	19q13.3	0.0076	0.4551
actin related protein 2/3 complex, subunit 5, 16kDa	ARPC5	Hs.126222	1q25.3	0.0002	0.4577
TBC1 domain family, member 15	TBC1D15	Hs.310422	12q21.1	0.0002	0.0485
Transcribed sequences	EST	Hs.164221		0.0028	0.9374
mitogen-activated protein kinase 10	MAPK10	Hs.25209	4q22.1-q23	0.0003	0.0039

fibrinogen, B beta polypeptide	FGB	Hs.300774	4q28	0.0082	0.0366
calcium/calmodulin-dependent protein kinase II	CaMKIINalpha	Hs.197922	1p36.12	0.0039	0.0042
Data not found	EST	Hs.133262		0.0000	0.0342
flavin containing monooxygenase 3	FMO3	Hs.80876	1q23-q25	0.0020	0.3632
calcium/calmodulin-dependent protein kinase (CaM ki	CAMK2G	Hs.12436	10q22	0.0058	0.1560
myosin, light polypeptide 4, alkali; atrial, embryonic	MYL4	Hs.356717	17q21-qter	0.0094	0.3632
hypothetical protein PRO1580	PRO1580	Hs.270863	5q31.3	0.0079	1.0000
Transcribed sequence with strong similarity to protein	EST	Hs.527260		0.0075	0.0683
G protein-coupled receptor 86	GPR86	Hs.13040	3q24	0.0085	0.2186
paraspeckle component 1	PSPC1	Hs.16364	13q12.11	0.0039	0.0443
Transcribed sequence with strong similarity to protein	EST	Hs.320018		0.0002	0.1417
hypothetical protein BC009073	LOC90050	Hs.112654	14q32.13	0.0000	0.3632
Similar to 40S ribosomal protein SA (P40) (34/67 kDa	EST	Hs.448968	19p12	0.0001	0.0015
fetal Alzheimer antigen	FALZ	Hs.440947	17q24.3	0.0074	0.0018
alpha-2-HS-glycoprotein	AHSG	Hs.324746	3q27	0.0047	0.0068
hypothetical protein FLJ31842	FLJ31842	Hs.84522	1p21.3	0.0022	0.0005
DnaJ (Hsp40) homolog, subfamily C, member 3	DNAJC3	Hs.6019		0.0027	0.0617
tRNA isopentenyltransferase 1	TRIT1	Hs.356554	1p35.3-p34.1	0.0004	0.0281
Wilms tumor 1	WT1	Hs.1145	11p13	0.0008	0.0824
zinc finger protein 226	ZNF226	Hs.145956	19q13.2	0.0057	0.1521
transcriptional regulating factor 1	TRERF1	Hs.50102		0.0007	0.0117
family with sequence similarity 3, member C	FAM3C	Hs.434053	7q22.1-q31.1	0.0000	0.3632
histidine-rich glycoprotein	HRG	Hs.1498	3q27	0.0015	0.2984
arginine vasopressin receptor 1A	AVPR1A	Hs.2131	12q14-q15	0.0005	0.0493
serine protease inhibitor, Kazal type, 5	SPINK5	Hs.331555	5q32	0.0003	0.6628
major histocompatibility complex, class II, DQ beta 1	HLA-DQB1	Hs.409934	6p21.3	0.0043	0.4644
aortic preferentially expressed protein 1	APEG1	Hs.21639	2q35	0.0009	0.0204
Hypothetical gene supported by BC052560 (LOC3877	EST	Hs.448680		0.0000	0.0199
translocase of outer mitochondrial membrane 7 homol	TOMM7	Hs.112318	7p15.3	0.0000	0.2009
tumor necrosis factor (ligand) superfamily, member 1(	TNFSF10	Hs.387871	3q26	0.0021	0.7828
SA hypertension-associated homolog (rat)	SAH	Hs.512678	16p13.11	0.0009	0.1708
Partial mRNA for IgM immunoglobulin heavy chain v	EST	Hs.448936		0.0008	0.0455
Similar to Amine oxidase [flavin-containing] A (Monc	EST	Hs.522842		0.0053	0.0242
ADP-ribosylation factor 3	ARF3	Hs.119177	12q13	0.0002	0.7353
CCAAT/enhancer binding protein (C/EBP), alpha	CEBPA	Hs.76171	19q13.1	0.0018	0.1197
collapsin response mediator protein 1	CRMP1	Hs.155392	4p16.1-p15	0.0066	0.1777
chitinase 3-like 2	CHI3L2	Hs.154138	1p13.3	0.0039	0.3632
START domain containing 3	STARD3	Hs.77628	17q11-q12	0.0048	1.0000
nudE nuclear distribution gene E homolog like 1 (A. n	NDEL1	Hs.3850	17p13.1	0.0062	1.0000
baculoviral IAP repeat-containing 1	BIRC1	Hs.79019	5q13.1	0.0046	0.2043
lecithin-cholesterol acyltransferase	LCAT	Hs.387239	16q22.1	0.0000	0.1450
ATPase, H+ transporting, lysosomal 13kDa, V1 subun	ATP6V1G2	Hs.249227	6p21.3	0.0011	1.0000
ATP-binding cassette, sub-family D (ALD), member 1	ABCD1	Hs.159546	Xq28	0.0004	1.0000
serine (or cysteine) proteinase inhibitor, clade G (C1	SERPING1	Hs.384598	11q12-q13.1	0.0070	0.0093
Clone IMAGE:4249217, mRNA	EST	Hs.296141		0.0001	0.1756
clusterin (complement lysis inhibitor, SP-40,40, sulfat	CLU	Hs.436657	8p21-p12	0.0045	0.0705
chemokine (C-C motif) ligand 13	CCL13	Hs.414629	17q11.2	0.0005	0.0383
hypothetical protein LOC118987	LOC118987	Hs.131834	10q26.11	0.0085	0.3632
pim-1 oncogene	PIM1	Hs.81170	6p21.2	0.0082	0.1977
UDP glycosyltransferase 1 family, polypeptide A10	UGT1A10	Hs.278896		0.0004	0.0003
protein disulfide isomerase related protein (calcium-bi	ERP70	Hs.93659	7q35	0.0024	0.0256
jagged 1 (Alagille syndrome)	JAG1	Hs.409202	20p12.1-p11.23	0.0094	0.0321
phosphoribosyl pyrophosphate synthetase-associated p	PRPSAP2	Hs.13339	17p11.2-p12	0.0018	0.3632
APEX nuclease (multifunctional DNA repair enzyme)	APEX1	Hs.73722	14q11.2-q12	0.0058	0.3632
4-hydroxyphenylpyruvate dioxygenase	HPD	Hs.2899	12q24-qter	0.0029	0.0001
Human transglutaminase mRNA, 3' untranslated regio:	EST	Hs.458353		0.0002	0.3632
proteoglycan 1, secretory granule	PRG1	Hs.1908	10q22.1	0.0036	0.1388
phosphoserine aminotransferase 1	PSAT1	Hs.286049	9q21.2	0.0059	0.1830
glutamate dehydrogenase 2	GLUD2	Hs.525862	Xq24-q25	0.0034	0.0424
E4F transcription factor 1	E4F1	Hs.154196	16p13.3	0.0001	1.0000
TAR (HIV) RNA binding protein 2	TARBP2	Hs.326	12q12-q13	0.0037	0.3632

ADP-ribosylation factor 4	ARF4	Hs.435639	3p21.2-p21.1	0.0004	0.0063
threonyl-tRNA synthetase	TARS	Hs.84131	5p13.2	0.0053	0.0422
golgi-specific brefeldin A resistance factor 1	GBF1	Hs.155499	10q24	0.0035	0.0180
calnexin	CANX	Hs.155560	5q35	0.0001	0.0042
MCM3 minichromosome maintenance deficient 3 (S. c	MCM3	Hs.179565	6p12	0.0000	0.3632
General transcription factor II, i, pseudogene 1, mRNA	EST	Hs.169921		0.0062	0.1726
zinc ribbon domain containing, 1	ZNRD1	Hs.57813	6p21.3	0.0082	1.0000
chromosome 10 open reading frame 45	C10orf45	Hs.103378	10p13	0.0000	0.0791
solute carrier family 9 (sodium/hydrogen exchanger), i	SLC9A3R1	Hs.396783	17q25.1	0.0067	0.0067
KIAA0063 gene product	KIAA0063	Hs.3094	22q13.1	0.0075	0.1856
Jumonji, AT rich interactive domain 1C (RBP2-like)	JARID1C	Hs.103381	Xp11.22-p11.2	0.0020	0.0500
abl interactor 2	ABI2	Hs.387906	2q33	0.0001	0.0233
Phosphoserine phosphatase-like mRNA, complete cds	EST	Hs.490626		0.0005	0.3977
catenin (cadherin-associated protein), alpha-like 1	CTNNAL1	Hs.58488	9q31.2	0.0044	0.0058
plasminogen	PLG	Hs.143436	6q26	0.0009	0.7675
adenosine A2b receptor	ADORA2B	Hs.45743	17p12-p11.2	0.0056	1.0000
EphA7	EPHA7	Hs.73962	6q16.1	0.0039	0.2595
microtubule-associated protein 2	MAP2	Hs.167	2q34-q35	0.0013	0.3632
NADH dehydrogenase (ubiquinone) flavoprotein 1, 51	NDUFV1	Hs.7744	11q13	0.0071	0.2273
zinc finger protein 398	ZNF398	Hs.169452	7q36.1	0.0000	0.4642
dCMP deaminase	DCTD	Hs.76894	4q35.1	0.0097	0.3632
tumor rejection antigen (gp96) 1	TRA1	Hs.192374	12q24.2-q24.3	0.0023	0.0018
membrane component, chromosome 11, surface marke	M11S1	Hs.278672	11p13	0.0006	0.0779
thymidylate synthetase	TYMS	Hs.87491	18p11.32	0.0078	0.2728
transcription elongation factor A (SII), 1	TCEA1	Hs.78869	8q11.2	0.0037	0.0494
dolichyl-diphosphooligosaccharide-protein glycosyltra	DDOST	Hs.301882	1p36.1	0.0004	0.3632
MUF1 protein	MUF1	Hs.144941	1p34.1	0.0001	1.0000
cell division cycle 2, G1 to S and G2 to M	CDC2	Hs.334562	10q21.1	0.0029	0.0058
integrin beta 1 binding protein 1	ITGB1BP1	Hs.200666	2p25.2	0.0004	1.0000
galactosidase, alpha	GLA	Hs.69089	Xq22	0.0021	1.0000
glucosidase, beta; acid (includes glucosylceramidase)	GBA	Hs.282997	1q21	0.0065	0.3632
RIO kinase 3 (yeast)	RIOK3	Hs.209061	18q11.2	0.0083	0.4910
uroporphyrinogen decarboxylase	UROD	Hs.78601	1p34	0.0005	0.3632
leukocyte receptor cluster (LRC) member 4	LENG4	Hs.78768	19q13.4	0.0066	0.3632
retinitis pigmentosa GTPase regulator	RPGR	Hs.378949	Xp11.4	0.0013	1.0000
protein tyrosine phosphatase, receptor type, F	PTPRF	Hs.75216	1p34	0.0015	1.0000
clathrin, heavy polypeptide (Hc)	CLTC	Hs.187416	17q11-qter	0.0063	0.3632
interleukin enhancer binding factor 2, 45kDa	ILF2	Hs.75117	1q21.3	0.0000	0.0516
activating transcription factor 4 (tax-responsive enhan	ATF4	Hs.181243	22q13.1	0.0004	0.8299
glutaminyl-tRNA synthetase	QARS	Hs.79322	3p21.3-p21.1	0.0025	0.1187
exportin 1 (CRM1 homolog, yeast)	XPO1	Hs.157367	2p16	0.0006	0.0483
nucleolin	NCL	Hs.79110	2q12-qter	0.0062	0.8787
similar to RIKEN cDNA A430101B06 gene	MGC13017	Hs.293659	5q31.1	0.0003	0.4293
thyroid autoantigen 70kDa (Ku antigen)	G22P1	Hs.169744	22q13.2-q13.31	0.0032	0.3632
hypothetical protein MGC11061	MGC11061	Hs.66309	2p22.3	0.0007	0.2957
Data not found	EST	Hs.308680		0.0005	0.3500
histone deacetylase 1	HDAC1	Hs.88556	1p34	0.0029	0.9745
uracil-DNA glycosylase	UNG	Hs.78853	12q23-q24.1	0.0005	1.0000
glyceraldehyde-3-phosphate dehydrogenase	GAPD	Hs.169476		0.0078	0.0863
cytochrome c oxidase subunit VIII	COX8	Hs.433901	11q12-q13	0.0019	0.0337
Similar to eukaryotic translation initiation factor 3,	EST	Hs.433280	2p16.1	0.0015	0.1735
proteasome (prosome, macropain) subunit, beta type, 7	PSMB7	Hs.197071	9q34.11-q34.12	0.0031	0.0571
Similar to BLOCK 23 (LOC220717), mRNA	EST	Hs.448226		0.0027	0.4189
lamin A/C	LMNA	Hs.436441	1q21.2-q21.3	0.0002	0.0962
KIAA0652 gene product	KIAA0652	Hs.410092	11p11.2	0.0007	0.4246
ATP synthase, H+ transporting, mitochondrial F1 com	ATP5C1	Hs.155433	10p15.1	0.0015	0.3893
claudin 5 (transmembrane protein deleted in velocardi	CLDN5	Hs.110903	22q11.21	0.0003	0.0113
COX15 homolog, cytochrome c oxidase assembly pro	COX15	Hs.226581	10q24	0.0001	0.0020
insulin-like growth factor 2 (somatomedin A)	IGF2	Hs.349109		0.0033	0.1898
mesoderm specific transcript homolog (mouse)	MEST	Hs.440459	7q32	0.0033	0.0065
eukaryotic translation initiation factor 3, subunit 8,	EIF3S8	Hs.388163	16p11.2	0.0038	0.7234

RNA binding motif protein 10	RBM10	Hs.348276	Xp11.23	0.0001	0.4395
interferon gamma receptor 2 (interferon gamma transd	IFNGR2	Hs.409200	21q22.11	0.0002	0.0291
immediate early response 2	IER2	Hs.737	19p13.13	0.0022	0.0478
AD-003 protein	AD-003	Hs.284250	9q34.11	0.0074	0.4189
ribosomal protein L27	RPL27	Hs.405528	17q21.1-q21.2	0.0015	0.0209
thymidylate synthetase	TYMS	Hs.87491	18p11.32	0.0009	0.0712
succinate dehydrogenase complex, subunit A, flavoprc	SDHA	Hs.440475	5p15	0.0030	0.9241
protein phosphatase 4 (formerly X), catalytic subunit	PPP4C	Hs.2903	16p12-16p11	0.0097	0.0317
U5 snRNP-specific protein, 200-KD	U5-200KD	Hs.246112	2q11.2	0.0056	0.0842
polymerase (DNA directed), epsilon 3 (p17 subunit)	POLE3	Hs.108112	9q33	0.0011	0.1426
protective protein for beta-galactosidase (galactosiali	PPGB	Hs.118126	20q13.1	0.0016	0.0887
phospholipase D3	PLD3	Hs.257008	19q13.2	0.0018	0.3746
chromosome 7 open reading frame 24	C7orf24	Hs.444840	7p15-p14	0.0022	0.2163
hypothetical protein FLJ20519	FLJ20519	Hs.435461	1q21.2	0.0047	0.0378
hypothetical protein FLJ20254	FLJ20254	Hs.15356	2p23.3	0.0065	0.3304
dehydrogenase E1 and transketolase domain containin;	DHTKD1	Hs.501565	10p14	0.0004	0.9509
adenylosuccinate lyase	ADSL	Hs.75527	22q13.1	0.0006	0.2402
chromosome 21 open reading frame 56	C21orf56	Hs.381214	21q22.3	0.0000	0.1999
dihydrofolate reductase	DHFR	Hs.464813		0.0004	0.4851
mitochondrial ribosomal protein L51	MRPL51	Hs.55847	12p13.3-p13.1	0.0057	0.0482
hypothetical protein MGC2603	MGC2603	Hs.149305	1p36.11	0.0033	0.3895
retinoblastoma binding protein 4	RBBP4	Hs.16003	1p35.1	0.0005	0.1187
hypothetical protein FLJ10597	FLJ10597	Hs.90375	1p34.1	0.0054	0.2942
ADP-ribosylation factor-like 2 binding protein	ARL2BP	Hs.9552	16q13	0.0004	0.6694
small nuclear ribonucleoprotein polypeptide G	SNRPG	Hs.436656		0.0067	0.0011
hydroxysteroid (17-beta) dehydrogenase 4	HSD17B4	Hs.356894	5q21	0.0014	0.8628
H2A histone family, member V	H2AV	Hs.301005	7p13	0.0030	0.0227
insulin-like growth factor binding protein 3	IGFBP3	Hs.450230	7p13-p12	0.0058	0.0132
proteasome (prosome, macropain) 26S subunit, non-A'	PSMD13	Hs.279554	11p15.5	0.0067	0.3632
A kinase (PRKA) anchor protein 1	AKAP1	Hs.78921	17q21-q23	0.0004	0.7900
hairy/enhancer-of-split related with YRPW motif 1	HEY1	Hs.234434	8q21	0.0028	0.2770
bifunctional apoptosis regulator	BFAR	Hs.435556	16p13.12	0.0007	0.7663
uroporphyrinogen decarboxylase	UROD	Hs.78601	1p34	0.0028	0.4628
protein phosphatase methylesterase-1	PME-1	Hs.63304	11q13.4	0.0065	0.0208
NADH dehydrogenase (ubiquinone) 1 alpha subcompl	NDUFA7	Hs.19561	19p13.2	0.0009	0.0012
Rab geranylgeranyltransferase, alpha subunit	RABGGTA	Hs.377992	14q11.2	0.0029	0.6974
MCM3 minichromosome maintenance deficient 3 (S. (	MCM3	Hs.179565	6p12	0.0097	0.0079
UDP-glucose pyrophosphorylase 2	UGP2	Hs.417361	2p14-p13	0.0015	0.0185
ribophorin II	RPN2	Hs.406532	20q12-q13.1	0.0001	0.0107
inhibitor of DNA binding 3, dominant negative helix-l	ID3	Hs.76884	1p36.13-p36.12	0.0029	0.2039
chromosome 7 open reading frame 20	C7orf20	Hs.107387	7p22.3	0.0068	0.2108
proliferation-associated 2G4, 38kDa	PA2G4	Hs.374491	12q13	0.0024	0.2208
FK506 binding protein 1A, 12kDa	FKBP1A	Hs.374638		0.0045	0.1836
NICE-4 protein	NICE-4	Hs.8127	1q21.3	0.0001	0.0561
ribosomal protein S3	RPS3	Hs.387576	11q13.3-q13.5	0.0001	0.0430
KIAA1536 protein	KIAA1536	Hs.156667	12q13.13	0.0050	0.1444
likely ortholog of mouse membrane bound C2 domain	MBC2	Hs.8309	12q13.2	0.0037	0.0585
tubulin beta MGC4083	MGC4083	Hs.274398	18p11.21	0.0010	0.0151
v-erb-b2 erythroblastic leukemia viral oncogene homo	ERBB3	Hs.306251	12q13	0.0008	0.2690
prolactin regulatory element binding	PREB	Hs.279784	2p23.3	0.0086	0.4540
hypothetical protein similar to RNA-binding protein la	MGC10871	Hs.49994	11q13	0.0072	0.2370
Gardner-Rasheed feline sarcoma viral (v-fgr) oncogen	FGR	Hs.1422	1p36.2-p36.1	0.0054	0.8368
tubulin alpha 6	TUBA6	Hs.406578	12q12-q14	0.0008	0.0112
nudix (nucleoside diphosphate linked moiety X)-type 1	NUDT2	Hs.429142	9p13	0.0001	1.0000
ribosomal protein S16	RPS16	Hs.397609	19q13.1	0.0060	0.0080
SRY (sex determining region Y)-box 7	SOX7	Hs.213194		0.0000	0.1445
nucleostemin	NS	Hs.313544	3p21.1	0.0018	0.0665
Similar to tubulin, beta 5 (LOC92755), mRNA	EST	Hs.458472	8p12	0.0001	0.1179
proteasome (prosome, macropain) 26S subunit, ATPas	PSMC3	Hs.250758	11p12-p13	0.0000	0.1149
nucleolin	NCL	Hs.79110	2q12-qter	0.0047	0.0378
exonuclease 1	EXO1	Hs.47504	1q42-q43	0.0000	0.0394

KIAA0121 gene product	KIAA0121	Hs.155584	3p25.3	0.0040	0.1961
MEP50 protein	MEP50	Hs.446551	1p13.2	0.0023	0.3632
TAR (HIV) RNA binding protein 2	TARBP2	Hs.326	12q12-q13	0.0022	0.8380
cyclin-dependent kinase 4	CDK4	Hs.95577	12q14	0.0019	0.3632
hypothetical protein FLJ10579	FLJ10579	Hs.8055	15q15.1	0.0014	0.4907
zinc finger protein 551	ZNF551	Hs.184846	19q13.43	0.0009	0.2465
POM121 membrane glycoprotein (rat)	POM121	Hs.380964		0.0005	0.0053
GABA(A) receptor-associated protein like 1	GABARAPL1	Hs.336429	12p13.2	0.0003	0.4311
follicular lymphoma variant translocation 1	FVT1	Hs.74050	18q21.3	0.0000	0.2044
NPD007 protein	NPD007	Hs.7788	7q11.23	0.0066	0.3060
carboxypeptidase Z	CPZ	Hs.78068	4p16.1	0.0019	0.0302
myogenin (myogenic factor 4)	MYOG	Hs.2830	1q31-q41	0.0006	0.3632
CDNA clone MGC:13162 IMAGE:3010103, complete	EST	Hs.99081		0.0015	0.0002
NCK adaptor protein 1	NCK1	Hs.54589	3q21	0.0015	0.0313
glutamate dehydrogenase 2	GLUD2	Hs.525862	Xq24-q25	0.0040	0.3204
mesenchymal stem cell protein DSC92	NEUGRIN	Hs.124452	15q26.1	0.0002	0.2706
hypothetical protein MGC14425	MGC14425	Hs.122655		0.0025	0.3632
phospholipase C, gamma 2 (phosphatidylinositol-speci	PLCG2	Hs.512298	16q24.1	0.0001	0.1253
hypothetical protein MGC45731	MGC45731	Hs.381105	1p12	0.0049	0.0978
hypothetical protein LOC339903	LOC339903	Hs.146346	3p22.1	0.0066	0.0165
hypothetical protein FLJ13852	FLJ13852	Hs.165186	8q24.3	0.0000	0.2818
F-box only protein 7	FBXO7	Hs.5912	22q12-q13	0.0034	0.4692
Clone IMAGE:5169682, mRNA	EST	Hs.334714		0.0095	0.3632
chromosome 9 open reading frame 83	C9orf83	Hs.415534	9p13.3	0.0029	0.0037
metallothionein 2A	MT2A	Hs.418241	16q13	0.0000	0.0293
thiopurine S-methyltransferase	TPMT	Hs.377402	6p22.3	0.0021	0.0001
lipoyltransferase 1	LIPT1	Hs.112356	2q11.2	0.0010	0.0000
serum amyloid A4, constitutive	SAA4	Hs.512677	11p15.1-p14	0.0045	0.0284
fatty acid binding protein 3, muscle and heart (mamma	FABP3	Hs.112669	1p33-p32	0.0019	0.0020
PTD016 protein	LOC51136	Hs.30154	17q23.2	0.0042	0.1059
hemopexin	HPX	Hs.426485	11p15.5-p15.4	0.0011	0.0087
titin immunoglobulin domain protein (myotilin)	TTID	Hs.84665	5q31	0.0017	0.1735
dihydropyrimidine dehydrogenase	DPYD	Hs.1602	1p22	0.0080	0.0337
Data not found	EST			0.0018	0.0084
tryptophan 2,3-dioxygenase	TDO2	Hs.183671	4q31-q32	0.0003	0.0007
carboxypeptidase B2 (plasma, carboxypeptidase U)	CPB2	Hs.221926	13q14.11	0.0047	0.0070
fibrinogen-like 1	FGL1	Hs.107	8p22-p21.3	0.0009	0.0254
geminin, DNA replication inhibitor	GMNN	Hs.234896	6p22.2	0.0062	0.3632
Data not found	EST	Hs.334568		0.0094	0.0115
sterol carrier protein 2	SCP2	Hs.75760	1p32	0.0029	0.0310
histamine N-methyltransferase	HNMT	Hs.42151	2q22.1	0.0072	0.1779
villin 2 (ezrin)	VIL2	Hs.403997	6q25.2-q26	0.0024	0.0000
tubulin, alpha 1 (testis specific)	TUBA1	Hs.75318		0.0064	0.1103
Huntingtin interacting protein K	HYPK	Hs.511978	15q15.3	0.0075	0.2917
myo-inositol 1-phosphate synthase A1	ISYNA1	Hs.405873	19p13.11	0.0054	0.1328
CAP, adenylate cyclase-associated protein 1 (yeast)	CAP1	Hs.104125	1p34.2	0.0091	0.1369
tubulin, alpha, ubiquitous	K-ALPHA-1	Hs.446608	12q13.12	0.0039	0.0041
malate dehydrogenase 1, NAD (soluble)	MDH1	Hs.75375	2p13.3	0.0001	0.0560
retinoblastoma-associated factor 600	RBAF600	Hs.287616	1p36.13	0.0055	0.5754
integrin beta 4 binding protein	ITGB4BP	Hs.5215	20q12	0.0039	0.0338
melanocortin 1 receptor (alpha melanocyte stimulating	MC1R	Hs.435105	16q24.3	0.0007	0.0531
Data not found	EST	Hs.239307		0.0054	0.1315
tubulin, beta, 5	TUBB5	Hs.110837	19p13.3	0.0000	0.0174
tubulin, beta, 2	TUBB2	Hs.433615		0.0005	0.0229
mitochondrial intermediate peptidase	MIPEP	Hs.68583	13q12	0.0087	0.3632
dolichyl-diphosphooligosaccharide-protein glycosyltra	DDOST	Hs.301882	1p36.1	0.0065	0.3057
PAGE-5 protein	PAGE-5	Hs.245431	Xp11.22	0.0087	0.0874
spermidine/spermine N1-acetyltransferase 2	SAT2	Hs.10846	17p13.1	0.0035	0.9386
hypothetical protein FLJ20345	FLJ20345	Hs.408843	17q23.2	0.0004	0.3632
hydroxyprostaglandin dehydrogenase 15-(NAD)	HPGD	Hs.77348	4q34-q35	0.0009	0.2653
small inducible cytokine subfamily E, member 1 (endo	SCYE1	Hs.105656	4q24	0.0062	0.1868

testis-specific protein TSP-NY	TSP-NY	Hs.97643	12q24.31	0.0013	0.6533
polymerase (RNA) III (DNA directed) polypeptide K,	POLR3K	Hs.437186	16p13.3	0.0060	0.3129
cerebroside (3'-phosphoadenylylsulfate:galactosylcerar	CST	Hs.17958	22q12.2	0.0058	0.3770
major histocompatibility complex, class II, DQ beta 1	HLA-DQB1	Hs.409934	6p21.3	0.0002	0.0835
hypothetical protein MGC20255	MGC20255	Hs.437497	19q13.2	0.0014	0.4195
small nuclear RNA activating complex, polypeptide 1,	SNAPC1	Hs.179312	14q22	0.0069	0.2722
tyrosine kinase 2	TYK2	Hs.75516	19p13.2	0.0028	0.2138
chromosome 8 open reading frame 4	C8orf4	Hs.283683	8p11.2	0.0054	0.0746
arginase, liver	ARG1	Hs.440934	6q23	0.0002	0.0185
RNA polymerase III subunit RPC8	RPC8	Hs.202505	22q13.2	0.0026	0.0620
asialoglycoprotein receptor 2	ASGR2	Hs.1259	17p	0.0029	0.0253
Transcribed sequence with moderate similarity to prot	EST	Hs.447471		0.0066	0.0268
G-protein signalling modulator 1 (AGS3-like, C. elega	GPSM1	Hs.239370	9q34.3	0.0023	0.0132
zinc finger protein 481	ZNF481	Hs.5638	9q33.2	0.0079	0.5210
mutS homolog 2, colon cancer, nonpolyposis type 1 (I	MSH2	Hs.440394	2p22-p21	0.0054	0.0230
hypothetical protein HSPC138	HSPC138	Hs.283322	11q14.2	0.0035	0.3417
pleiomorphic adenoma gene-like 2	PLAGL2	Hs.154104	20q11.21	0.0017	0.6277
troponin I, skeletal, fast	TNNI2	Hs.83760	11p15.5	0.0000	1.0000
malic enzyme 1, NADP(+)-dependent, cytosolic	ME1	Hs.14732	6q12	0.0032	0.0601
regulator of G-protein signalling 5	RGS5	Hs.24950	1q23.1	0.0074	0.0220
4-hydroxyphenylpyruvate dioxygenase	HPD	Hs.2899	12q24-qter	0.0000	0.0055
dual-specificity tyrosine-(Y)-phosphorylation regulate	DYRK1B	Hs.130988	19q12-13.1	0.0021	1.0000
phosphoenolpyruvate carboxykinase 1 (soluble)	PCK1	Hs.1872	20q13.31	0.0022	0.0678
popeye domain containing 3	POPDC3	Hs.458336	6q21	0.0043	0.3003
tubulin, beta, 2	TUBB2	Hs.433615		0.0027	0.2332
histatin 1	HTN1	Hs.250959	4q13	0.0003	0.5349
zinc finger protein (C2H2 type) 277	ZNF277	Hs.42636	7q31.1	0.0006	0.5699
chromosome 6 open reading frame 165	C6orf165	Hs.82921	6q15	0.0005	0.3632
ficolin (collagen/fibrinogen domain containing) 3 (Ha	FCN3	Hs.333383	1p36.11	0.0048	0.1417
intelectin 1 (galactofuranose binding)	ITLN1	Hs.50813		0.0042	1.0000
Data not found	EST	Hs.2388		0.0004	0.1472
transthyretin (prealbumin, amyloidosis type I)	TTR	Hs.427202	18q12.1	0.0008	0.0251
sulfotransferase family, cytosolic, 2A, dehydroepiandr	SULT2A1	Hs.81884	19q13.3	0.0062	0.2980
complement component 9	C9	Hs.1290	5p14-p12	0.0003	0.0000
fibrinogen, A alpha polypeptide	FGA	Hs.351593	4q28	0.0014	0.0135
methylmalonyl CoA epimerase	MCEE	Hs.94949	2p13.3	0.0020	0.0058
ficolin (collagen/fibrinogen domain containing) 1	FCN1	Hs.440898	9q34	0.0000	0.3632
cytochrome P450, family 2, subfamily C, polypeptide	CYP2C8	Hs.282871	10q23.33	0.0055	0.0294
pre-B-cell colony enhancing factor 1	PBEF1	Hs.293464	7q22.3	0.0013	0.0469
metallothionein 1G	MT1G	Hs.433391	16q13	0.0000	0.0112
Opa-interacting protein 2	OIP2	Hs.274170	13q13.1	0.0037	0.3150
metallothionein 1F (functional)	MT1F	Hs.438737	16q13	0.0000	0.0001
hypothetical protein FLJ23235	FLJ23235	Hs.211501	4p14	0.0000	0.0075
hypothetical protein MGC27345	MGC27345	Hs.511404	7q32.2	0.0001	0.0090
zinc finger protein 519	ZNF519	Hs.352635	18p11.21	0.0000	0.0007
solute carrier family 38, member 2	SLC38A2	Hs.298275	12q	0.0010	0.0031
metallothionein 1A (functional)	MT1A	Hs.440939	16q13	0.0000	0.0001
deoxyribonuclease I	DNASE1	Hs.436928	16p13.3	0.0013	0.1212
ATPase, H+ transporting, lysosomal 56/58kDa, V1 sul	ATP6V1B1	Hs.64173	2p13.1	0.0008	0.1816
RNA (guanine-9-) methyltransferase domain containin	RG9MTD1	Hs.57898	3q12.3	0.0023	0.0845
Transcribed sequence with strong similarity to protein	EST	Hs.480552		0.0094	0.0175
solute carrier family 22 (organic cation transporter),	SLC22A13	Hs.225941	3p21.3	0.0007	0.0102
cyclin-dependent kinase 4	CDK4	Hs.95577	12q14	0.0068	0.3961
ribophorin II	RPN2	Hs.406532	20q12-q13.1	0.0014	0.1719
tubulin beta MGC4083	MGC4083	Hs.274398	18p11.21	0.0001	0.3798
CDNA clone IMAGE:3628701, partial cds	EST	Hs.98028		0.0001	0.9644
Similar to tubulin, beta 5 (LOC92755), mRNA	EST	Hs.458472	8p12	0.0001	0.0903
very low density lipoprotein receptor	VLDLR	Hs.370422	9p24	0.0070	0.3632
Data not found	EST	Hs.166697		0.0076	0.2314
homeo box A4	HOXA4	Hs.147747	7p15-p14	0.0006	0.3675
CDC20 cell division cycle 20 homolog (S. cerevisiae)	CDC20	Hs.82906	1p34.1	0.0032	0.3632

CDC20 cell division cycle 20 homolog (S. cerevisiae)	CDC20	Hs.82906	1p34.1	0.0015	0.3632
hypothetical protein LOC51244	LOC51244	Hs.236257	3p25.1	0.0060	0.0009
chemokine (C-X-C motif) ligand 2	CXCL2	Hs.75765	4q21	0.0062	0.0009
solute carrier family 35, member A4	SLC35A4	Hs.173103	5q31.3	0.0052	0.3303
Transcribed sequence with moderate similarity to prot	EST	Hs.483430		0.0091	0.0281
Transcribed sequence with strong similarity to protein	EST	Hs.450326		0.0015	0.0660
ribosomal protein L12	RPL12	Hs.408054		0.0002	0.1443
CDNA FLJ42958 fis, clone BRSTN2010750	EST	Hs.41688		0.0012	0.2763
PIN2-interacting protein 1	PINX1	Hs.400259	8p23	0.0016	0.0033
Clone IMAGE:3616855, mRNA	EST	Hs.225083		0.0064	0.0101
guanine nucleotide binding protein-like 1	GNL1	Hs.83147	6p21.3	0.0036	0.6147
PP1201 protein	PP1201	Hs.434007	2p24.3-p24.1	0.0040	0.1675
hypothetical protein FLJ13852	FLJ13852	Hs.165186	8q24.3	0.0083	0.1955
hypothetical protein LOC157567	LOC157567	Hs.11506		0.0055	0.1231
KIAA1102 protein	KIAA1102	Hs.156761	4p13	0.0006	0.1291
bone morphogenetic protein 1	BMP1	Hs.1274	8p21	0.0013	0.4172
Transcribed sequence with strong similarity to protein	EST	Hs.478508		0.0092	0.2662
Transcribed sequence with strong similarity to protein	EST	Hs.460344		0.0000	0.1092
chromosome 6 open reading frame 83	C6orf83	Hs.284265	6q27	0.0008	0.1429
ubiquitin-protein isopeptide ligase (E3)	KIAA0010	Hs.155287	7q36.3	0.0034	0.0169
LOC89231	LOC89231	Hs.487918		0.0005	0.2493
arginyl aminopeptidase (aminopeptidase B)	RNPEP	Hs.283667	1q32	0.0007	0.0363
interleukin 1 receptor, type II	IL1R2	Hs.25333	2q12-q22	0.0025	0.4657
dihydroorotate dehydrogenase	DHODH	Hs.405639	16q22	0.0021	0.2008
zinc finger and BTB domain containing 1	ZBTB1	Hs.511938	14q23.3	0.0051	0.1225
RNA polymerase I associated factor 53	PAF53	Hs.24884	9p13.2	0.0001	0.5640
CDNA FLJ27497 fis, clone TST05436	EST	Hs.205439		0.0011	0.0142
LOC400602 (LOC400602), mRNA	EST	Hs.408434		0.0014	0.0028
cytochrome P450, family 19, subfamily A, polypeptid	CYP19A1	Hs.187471	15q21.1	0.0075	0.0389
golgi reassembly stacking protein 2, 55kDa	GORASP2	Hs.6880	2q31.1-q31.2	0.0096	0.0049
mitochondrial tumor suppressor gene 1	MTSG1	Hs.7946	8p22	0.0003	0.0373
tyrosine aminotransferase	TAT	Hs.161640	16q22.1	0.0017	0.2321
coagulation factor XI (plasma thromboplastin antecede	F11	Hs.1430	4q35	0.0027	0.4032
mannosidase, alpha, class 1A, member 2	MAN1A2	Hs.367638	1p13	0.0046	0.0230
hypothetical protein DKFZp762C1112	DKFZp762C11	Hs.88594	8q21.3	0.0022	0.1442
Transcribed sequence with weak similarity to protein r	EST	Hs.3731		0.0061	0.1544
chromosome 10 open reading frame 76	C10orf76	Hs.9444	10q24.32	0.0049	0.0257
ATP synthase mitochondrial F1 complex assembly fac	ATPAF2	Hs.13434	17p11.2	0.0035	0.3632
cell division cycle 25C	CDC25C	Hs.656	5q31	0.0000	0.0212
aldolase A, fructose-bisphosphate	ALDOA	Hs.273415	16q22-q24	0.0058	0.5172
novel 58.3 KDA protein	LOC91614	Hs.180545	11p13	0.0000	0.6823
ceruloplasmin (ferroxidase)	CP	Hs.282557	3q23-q25	0.0001	0.0300
Clone IMAGE:4671447, mRNA	EST	Hs.382687		0.0001	0.1032
Transcribed sequence with moderate similarity to prot	EST	Hs.447471		0.0062	0.0061
KIAA0692 protein	KIAA0692	Hs.524874	12q24.33	0.0037	0.0581
peptidase (mitochondrial processing) alpha	PMPCA	Hs.75353	9q34.3	0.0100	0.6792
ribosomal protein S3	RPS3	Hs.387576	11q13.3-q13.5	0.0001	0.0244
Transcribed sequence with moderate similarity to prot	EST	Hs.461408		0.0013	0.8382
Clone IMAGE:5015646, mRNA	EST	Hs.385483		0.0001	0.1282
endoglycan	PODLX2	Hs.145416	15	0.0012	0.8763
C-terminal binding protein 1	CTBP1	Hs.196083	4p16	0.0001	0.0731
antigen p97 (melanoma associated) identified by mon	MFI2	Hs.252855	3q28-q29	0.0085	0.4161
methyltransferase like 3	METTL3	Hs.168799	14q11.1	0.0044	0.3632
IKK interacting protein	IKIP	Hs.406199	12q23.1	0.0008	0.0880
Clone BAC 33J7 diphosphoinositol polyphosphate ph	EST	Hs.356699		0.0049	0.2281
sema domain, immunoglobulin domain (Ig), transmem	SEMA4G	Hs.444359	10q24.32	0.0018	0.3632
Similar to tubulin, beta 5 (LOC92755), mRNA	EST	Hs.458472	8p12	0.0003	0.0260
Clone IMAGE:6646977, mRNA	EST	Hs.162880		0.0000	0.9022
Transcribed sequence with moderate similarity to prot	EST	Hs.461412		0.0001	0.1556
zinc finger protein 584	ZNF584	Hs.439551	19q13.43	0.0058	0.1521
exportin 1 (CRM1 homolog, yeast)	XPO1	Hs.157367	2p16	0.0001	0.3566

hypothetical protein FLJ10851	FLJ10851	Hs.17860	10q11.23	0.0001	1.0000
glyceraldehyde-3-phosphate dehydrogenase, spermato	GAPDS	Hs.248017	19q13.1	0.0062	1.0000
nardilysin (N-arginine dibasic convertase)	NRD1	Hs.4099	1p32.2-p32.1	0.0009	0.5334
glyceraldehyde-3-phosphate dehydrogenase	GAPD	Hs.169476		0.0056	0.1446
Hypothetical protein LOC339894 (LOC339894), mRN	EST	Hs.398203		0.0006	0.0333
CDNA FLJ41467 fis, clone BRSTN2017995	EST	Hs.147878		0.0027	0.1882
kelch-like 11 (Drosophila)	KLHL11	Hs.13268	17q21.2	0.0024	0.0233
actin related protein 2/3 complex, subunit 3, 21kDa	ARPC3	Hs.439511	12q24.11	0.0060	0.4203
5,10-methenyltetrahydrofolate synthetase (5-formylteti	MTHFS	Hs.118131	15q25.1	0.0030	0.3632
Clone IMAGE:4826263, mRNA	EST	Hs.250839		0.0000	0.6285
zinc finger, DHHC domain containing 21	ZDHHC21	Hs.21388	9p22.3	0.0003	0.7103
Clone IMAGE:4824668, mRNA	EST	Hs.385720		0.0002	0.3620
Clone IMAGE:4823013, mRNA	EST	Hs.400608		0.0046	0.0959
leukotriene A4 hydrolase	LTA4H	Hs.81118	12q22	0.0010	0.3632
zinc finger protein 92 (HTF12)	ZNF92	Hs.9521	7q11.21	0.0075	0.0256
hypothetical protein MAC30	MAC30	Hs.199695	17q11.2	0.0046	0.1019
Similar to ribosomal protein L13a; 60S ribosomal prot	EST	Hs.446637	13q14.3	0.0044	0.3632
stathmin 1/oncoprotein 18	STMN1	Hs.209983	1p36.1-p35	0.0037	0.1880
MRNA; cDNA DKFZp686C135 (from clone DKFZp6	EST	Hs.504592		0.0005	0.3632
Similar to hypothetical protein LOC163227, clone IM	EST	Hs.428579		0.0098	0.4282
Clone IMAGE:5294815, mRNA	EST	Hs.385636		0.0000	0.3632
hypothetical protein MGC33993	MGC33993	Hs.111164	6p23	0.0094	0.7922
Data not found	EST	Hs.511920		0.0013	0.7048
neural cell adhesion molecule 2	NCAM2	Hs.135892	21q21.1	0.0001	0.7976
hypothetical protein MGC48625	MGC48625	Hs.20506	19q13.42	0.0005	1.0000
hypothetical protein DKFZp313M0720	DKFZp313M07	Hs.410941	12q12	0.0005	0.5539
similar to alpha tubulin	LOC112714	Hs.433336	2q21.1	0.0041	0.1283
tumor protein p53 binding protein, 1	TP53BP1	Hs.440968	15q15-q21	0.0008	1.0000
phosphatidylinositol 4-kinase, catalytic, beta polypept	PIK4CB	Hs.154846	1q21	0.0014	0.3632
activating transcription factor 4 (tax-responsive enhan	ATF4	Hs.181243	22q13.1	0.0002	0.3669
apoptosis related protein APR-3	38080	Hs.9527	2p23.3	0.0083	0.3632
tubulin, beta polypeptide	TUBB	Hs.512712	6p25	0.0069	0.2335
tubulin, alpha 8	TUBA8	Hs.137400	22q11.1	0.0037	0.0430
Clone IMAGE:5296353, mRNA	EST	Hs.34068		0.0003	0.3632
ADP-ribosylation factor 4	ARF4	Hs.435639	3p21.2-p21.1	0.0002	0.2077
Transcribed sequence with strong similarity to protein	EST	Hs.486676		0.0086	0.0255
secretory carrier membrane protein 3	SCAMP3	Hs.200600	1q21	0.0052	0.3505
Transcribed sequence with strong similarity to protein	EST	Hs.447673		0.0070	0.3632
heterogeneous nuclear ribonucleoprotein C (C1/C2)	HNRPC	Hs.476302	14q11.2	0.0023	0.3391
splicing factor, arginine/serine-rich 3	SFRS3	Hs.405144	6p21	0.0011	0.3632
peptidyl prolyl isomerase H (cyclophilin H)	PPIH	Hs.9880	1p34.1	0.0065	1.0000
melanoma antigen, family A, 12	MAGEA12	Hs.169246	Xq28	0.0002	0.4592
sulfotransferase family, cytosolic, 1A, phenol-preferri	SULT1A1	Hs.142	16p12.1	0.0001	0.5722
sulfotransferase family, cytosolic, 1A, phenol-preferri	SULT1A1	Hs.142	16p12.1	0.0005	0.0068
transcription elongation factor A (SII)-like 1	TCEAL1	Hs.95243	Xq22.1	0.0009	0.1837
KIAA1068 protein	KIAA1068	Hs.4770	7p13-p12	0.0018	0.4491
ribosomal protein S13	RPS13	Hs.446588	11p15	0.0010	0.0037
DKFZP586A0522 protein	DKFZP586A05	Hs.288771	12q13.12	0.0028	0.9552
mitochondrial ribosomal protein L15	MRPL15	Hs.18349	8q11.2-q13	0.0018	0.0271
epithelial cell transforming sequence 2 oncogene	ECT2	Hs.293257	3q26.1-q26.2	0.0070	0.3632
PRO0659 protein	PRO0659	Hs.460663		0.0053	0.1197
hypoxia-inducible protein 2	HIG2	Hs.61762	7q32.2	0.0024	0.0680
hypothetical protein MGC5576	MGC5576	Hs.103834	12q13.1	0.0083	0.3664
galactose-4-epimerase, UDP-	GALE	Hs.76057	1p36-p35	0.0014	0.3632
dCMP deaminase	DCTD	Hs.76894	4q35.1	0.0076	0.3632
tubulin, alpha, ubiquitous	K-ALPHA-1	Hs.446608	12q13.12	0.0048	0.0836
SWI/SNF related, matrix associated, actin dependent r	SMARCC2	Hs.236030	12q13-q14	0.0003	0.8371
zinc finger protein 22 (KOX 15)	ZNF22	Hs.108642	10q11	0.0000	0.0710
metallothionein 2A	MT2A	Hs.418241	16q13	0.0000	0.0015
phosphorylase, glycogen; brain	PYGB	Hs.145820	20p11.2-p11.1	0.0020	0.0106
chromosome 20 open reading frame 64	C20orf64	Hs.440263	20q13.2	0.0003	0.0035

NADH dehydrogenase (ubiquinone) 1 beta subcomple	NDUFB1	Hs.183435	14q32.12	0.0041	0.0009
coiled-coil domain containing 7	CCDC7	Hs.407833		0.0001	0.0178
hypothetical protein PRO1580	PRO1580	Hs.270863	5q31.3	0.0088	0.1869
putative small membrane protein NID67	NID67	Hs.29444	5q33.1	0.0056	0.3632
ATP-binding cassette, sub-family D (ALD), member 3	ABCD3	Hs.76781	1p22-p21	0.0025	0.3801
hypothetical protein FLJ20445	FLJ20445	Hs.343748	10q23.32-q23.3	0.0070	0.0088
erythroid differentiation-related factor 1	EDRF1	Hs.355929	10q26.13-q26.2	0.0075	0.3632
chromosome 14 open reading frame 130	C14orf130	Hs.6877	14q32.13	0.0075	0.0824
melanoma antigen, family A, 1 (directs expression of a	MAGEA1	Hs.72879	Xq28	0.0003	0.2240
Ig superfamily protein	Z39IG	Hs.8904	Xq12-q13.3	0.0092	1.0000
Clone IMAGE:4151631, mRNA	EST	Hs.350552		0.0011	0.0065
serine (or cysteine) proteinase inhibitor, clade G (C1	SERPING1	Hs.384598	11q12-q13.1	0.0001	0.0108
K+ channel tetramerization protein	GMRP-1	Hs.332382	11p15.2	0.0012	0.0047
queuine tRNA-ribosyltransferase 1 (tRNA-guanine tra	QTRT1	Hs.323084	19p13.3	0.0005	0.0921
chromosome 3 open reading frame 10	C3orf10	Hs.421654	3p25.3	0.0011	0.9263
proteoglycan 1, secretory granule	PRG1	Hs.1908	10q22.1	0.0029	0.0573
TEA domain family member 4	TEAD4	Hs.94865	12p13.2-p13.3	0.0072	1.0000
X-linked protein	DJ79P11.1	Hs.398989	Xq22	0.0078	0.3180
procollagen-lysine, 2-oxoglutarate 5-dioxygenase (lysi	PLOD	Hs.75093	1p36.3-p36.2	0.0064	0.3632
taxol resistance associated gene 3	TRAG3	Hs.458484	Xq28	0.0015	0.3632
melanoma antigen, family A, 2	MAGEA2	Hs.36980	Xq28	0.0041	1.0000
granulin	GRN	Hs.180577	17q21.32	0.0049	0.3632
homeo box C11	HOXC11	Hs.127562	12q13.3	0.0002	1.0000
asp (abnormal spindle)-like, microcephaly associated (	ASPM	Hs.121028	1q31	0.0088	0.5421
nucleolar and spindle associated protein 1	NUSAP1	Hs.279905	15q15.1	0.0000	0.1065
ubiquitin specific protease 24	USP24	Hs.7243	1p32.3	0.0020	1.0000
hypothetical protein MGC34831	MGC34831	Hs.367964	Xp21.1	0.0000	0.0628
H1 histone family, member 0	H1F0	Hs.226117	22q13.1	0.0001	0.1271
serine (or cysteine) proteinase inhibitor, clade F (alp	SERPINF2	Hs.159509	17p13	0.0009	0.0685
ribosomal protein L27	RPL27	Hs.405528	17q21.1-q21.2	0.0001	0.0215
TAP binding protein (tapasin)	TAPBP	Hs.370937	6p21.3	0.0004	0.1172
thyroid autoantigen 70kDa (Ku antigen)	G22P1	Hs.169744	22q13.2-q13.31	0.0017	0.2876
ribophorin I	RPN1	Hs.2280		0.0001	0.1158
protein kinase, cAMP-dependent, regulatory, type I, al	PRKAR1A	Hs.280342	17q23-q24	0.0026	0.3037
complement component 4A	C4A	Hs.150833		0.0053	0.2802
carboxylesterase 1 (monocyte/macrophage serine ester	CES1	Hs.278997	16q13-q22.1	0.0035	0.0925
Transcribed sequence with strong similarity to protein	EST	Hs.490146		0.0091	0.3360
CGG triplet repeat binding protein 1	CGGBP1	Hs.444818	3p12-p11.1	0.0010	0.5346
CDNA clone IMAGE:4156718, partial cds	EST	Hs.382184		0.0005	0.3632
catalase	CAT	Hs.395771	11p13	0.0018	0.0655
fms-related tyrosine kinase 4	FLT4	Hs.415048		0.0057	0.1744
protein tyrosine phosphatase, receptor type, A	PTPRA	Hs.306676		0.0033	0.1072
Clone IMAGE:4420333, mRNA	EST	Hs.382039		0.0075	1.0000
Clone IMAGE:3923185, mRNA	EST	Hs.354068		0.0069	0.0878
guanine nucleotide binding protein (G protein), beta po	GNB2L1	Hs.5662	5q35.3	0.0001	0.2035
mannosyl (alpha-1,3-)-glycoprotein beta-1,4-N-acetylg	MGAT4B	Hs.437277	5q35	0.0082	1.0000
major histocompatibility complex, class II, DQ beta 1	HLA-DQB1	Hs.409934	6p21.3	0.0010	1.0000
hypothetical protein FLJ22595	FLJ22595	Hs.287702	3q25.33	0.0014	0.3632
Clone IMAGE:4753178, mRNA	EST	Hs.382631		0.0059	0.4092
transforming growth factor, beta receptor I (activin A	TGFBR1	Hs.28005	9q22	0.0055	0.3632
Data not found	EST	Hs.0		0.0000	0.1204
insulin-like growth factor binding protein 1	IGFBP1	Hs.401316	7p13-p12	0.0002	0.0070
insulin-like growth factor binding protein 1	IGFBP1	Hs.401316	7p13-p12	0.0001	0.0214
clusterin (complement lysis inhibitor, SP-40,40, sulfat	CLU	Hs.436657	8p21-p12	0.0010	0.1723
golgi membrane protein SB140	SMAP-5	Hs.5672	5q31.3	0.0028	0.4577
myeloid inhibitory C-type lectin-like receptor	MICL	Hs.190519	12p13.31	0.0083	0.4573
sulfotransferase family, cytosolic, 1A, phenol-preferri	SULT1A2	Hs.389536	16p12.1	0.0003	0.0448
DNA-damage-inducible transcript 4	DDIT4	Hs.111244	10pter-q26.12	0.0000	0.8454
metallothionein 1X	MT1X	Hs.374950	16q13	0.0001	0.0008
chromosome 14 open reading frame 28	C14orf28	Hs.82098	14q21.2	0.0059	0.4494
glucosidase, beta; acid (includes glucosylceramidase)	GBA	Hs.282997	1q21	0.0009	1.0000

succinate dehydrogenase complex, subunit C, integral	SDHC	Hs.433982	1q21	0.0000	0.3632
metallothionein 1K	MT1K	Hs.188518	16q13	0.0000	0.0079
Clone IMAGE:5209417, mRNA	EST	Hs.171192		0.0003	0.0813
mitogen-activated protein kinase kinase 3	MAP2K3	Hs.180533	17q11.2	0.0012	0.0417
PR domain containing 10	PRDM10	Hs.275086	11q25	0.0046	0.0003
activin A receptor, type I	ACVR1	Hs.150402	2q23-q24	0.0028	0.1344
hypothetical protein FLJ31121	FLJ31121	Hs.350194	5q31.3	0.0099	0.1066
hypothetical protein MGC45491	MGC45491	Hs.149532	6p21.1	0.0089	0.3632
ribosomal protein L7a	RPL7A	Hs.416801	9q34	0.0001	0.0553
leukotriene A4 hydrolase	LTA4H	Hs.81118	12q22	0.0048	0.3632
synaptotagmin binding, cytoplasmic RNA interacting	SYNCRIP	Hs.436376		0.0025	0.2083
voltage-dependent calcium channel gamma subunit-lik	PR1	Hs.26216	2q14.2	0.0046	0.9455
vav 2 oncogene	VAV2	Hs.4248	9q34.1	0.0054	1.0000
flavoprotein oxidoreductase MICAL3	MICAL3	Hs.165551	22q11.21	0.0069	0.2218
Hypothetical protein LOC284593 (LOC284593), mRN	EST	Hs.399150		0.0000	0.0137
casein kinase 1, delta	CSNK1D	Hs.378918	17q25	0.0007	1.0000
syndecan 2 (heparan sulfate proteoglycan 1, cell surfac	SDC2	Hs.1501	8q22-q23	0.0009	0.1513
myosin, heavy polypeptide 9, non-muscle	MYH9	Hs.146550	22q13.1	0.0009	0.4945
progestin and adipoQ receptor family member X	PAQR10	Hs.379190	7p22.1	0.0033	0.1880
origin recognition complex, subunit 3-like (yeast)	ORC3L	Hs.312419	6q14.3-q16.1	0.0094	1.0000
timeless homolog (Drosophila)	TIMELESS	Hs.118631	12q12-q13	0.0015	1.0000
Similar to beta-tubulin 4Q (LOC286222), mRNA	EST	Hs.433616		0.0001	0.3632
beta 5-tubulin	OK/SW-cl.56	Hs.356729	6p21.33	0.0001	0.0409
Similar to tubulin, beta 5 (LOC92755), mRNA	EST	Hs.458472	8p12	0.0000	0.1587
tubulin, alpha, ubiquitous	K-ALPHA-1	Hs.446608	12q13.12	0.0033	0.0000
beta 5-tubulin	OK/SW-cl.56	Hs.356729	6p21.33	0.0001	0.0564
Transcribed sequences	EST	Hs.443097		0.0005	1.0000
unactive progesterone receptor, 23 kD	TEBP	Hs.355693	12q13.3	0.0003	0.0248
adenosylmethionine decarboxylase 1	AMD1	Hs.159118	6q21-q22	0.0014	0.3632
importin 13	IPO13	Hs.158497	1p34.1	0.0000	0.3632
polymerase (RNA) II (DNA directed) polypeptide A,	POLR2A	Hs.171880	17p13.1	0.0094	0.1249
stathmin 1/oncoprotein 18	STMN1	Hs.209983	1p36.1-p35	0.0090	0.0034
Clone IMAGE:4827547, mRNA	EST	Hs.436938		0.0047	0.3632

est)
